# Multi-Cellular Immunological Interactions Associated With COVID-19 Infections

**DOI:** 10.3389/fimmu.2022.794006

**Published:** 2022-02-24

**Authors:** Jitender S. Verma, Claudia R. Libertin, Yash Gupta, Geetika Khanna, Rohit Kumar, Balvinder S. Arora, Loveneesh Krishna, Folorunso O. Fasina, James B. Hittner, Athos Antoniades, Marc H. V. van Regenmortel, Ravi Durvasula, Prakasha Kempaiah, Ariel L. Rivas

**Affiliations:** ^1^Central Institute of Orthopaedics, Vardhman Mahavir Medical College and Safdarjung Hospital, New Delhi, India; ^2^Infectious Diseases, Mayo Clinic, Jacksonville, FL, United States; ^3^Respiratory Medicine, Vardhman Mahavir Medical College and Safdarjung Hospital, New Delhi, India; ^4^Department of Microbiology, Vardhman Mahavir Medical College and Safdarjung Hospital, New Delhi, India; ^5^Food and Agriculture Organization of the United Nations, Dar es Salaam, Tanzania; ^6^Department of Veterinary Tropical Diseases, University of Pretoria, Pretoria, South Africa; ^7^Psychology, College of Charleston, Charleston, SC, United States; ^8^Stremble Ventures LTD, Limassol, Cyprus; ^9^Medical University of Vienna, Vienna, Austria; ^10^Higher School of Biotechnology, University of Strasbourg, Strasbourg, France; ^11^Center for Global Health-Division of Infectious Diseases, School of Medicine, University of New Mexico, Albuquerque, NM, United States

**Keywords:** COVID-19, pattern recognition, cutoff-free, error prevention, biological complexity, personalized methods, multi-cellularity, personalized medicine

## Abstract

To rapidly prognosticate and generate hypotheses on pathogenesis, leukocyte multi-cellularity was evaluated in SARS-CoV-2 infected patients treated in India or the United States (152 individuals, 384 temporal observations). Within hospital (<90-day) death or discharge were retrospectively predicted based on the admission complete blood cell counts (CBC). Two methods were applied: (i) a “reductionist” one, which analyzes each cell type separately, and (ii) a “non-reductionist” method, which estimates multi-cellularity. The second approach uses a proprietary software package that detects distinct data patterns generated by complex and hypothetical indicators and reveals each data pattern’s immunological content and associated outcome(s). In the Indian population, the analysis of isolated cell types did not separate survivors from non-survivors. In contrast, multi-cellular data patterns differentiated six groups of patients, including, in two groups, 95.5% of all survivors. Some data structures revealed one data point-wide line of observations, which informed at a personalized level and identified 97.8% of all non-survivors. Discovery was also fostered: some non-survivors were characterized by low monocyte/lymphocyte ratio levels. When both populations were analyzed with the non-reductionist method, they displayed results that suggested survivors and non-survivors differed immunologically as early as hospitalization day 1.

## Introduction

The rapid extraction of more or new biologically interpretable information from the same data is a classic priority of clinical medicine and biomedical research. This goal is pursued by integrative approaches, which analyze several biological levels ‒including but not limited to genetic, molecular, cellular, and supra-cellular relationships (1, 2). For example, in COVID-19, mass cytometry has identified three disease phenotypes (3).

Yet, integrative biology still faces significant computational challenges (4). They derive from two competing needs. On the one hand, technologists feel pressed to “reduce dimensions” ‒so the time and cost involved in data analysis are reduced. Therefore, only “principal” data components are prioritized. This situation is driven by ‘the curse of dimensionality’: datasets may not be statistically treatable because there may be more parameters than data points (5). This operational emphasis is known as *reductionism*: it assumes that the whole is the sum of the parts. Consequently, inferences can be reduced to or explained by a few “low-level” variables (6, 7).

On the other hand, clinicians and researchers need biologically valid information fast, which should derive from and be applied to specific patients. This need applies to “*n*=1” situations, in which the number of patients is just one. While appropriate in population medicine, approaches that utilize averages are not applicable in personalized medicine (8). Furthermore, clinicians must address the complexities and dynamics of biological systems, such as those induced by host-pathogen-environmental relationships.

While reductionist techniques explicitly diminish the number of data inputs and, consequently, may miss information that characterizes system-level organizations, non-reductionist procedures do not do that. Instead, non-reductionist methods investigate distinct data patterns revealed by complex and dynamic biological systems (6, 7, 9–11). Is it possible to merge the strengths while limiting the weaknesses of bio-complexity and reductionism?

One approximation is to analyze multi-cellular interactions. While multi-cellularity is a well-known concept a structure composed of two or more cell types, the functions performed by groups of cells are less comprehended (12). It is now acknowledged that immune responses are not only determined by low-level structures (such as a single cell type or subtype) but also by groups of cells (13).

Immunological multi-cellular interactions may inform beyond network analysis. One example of such models is to capture one-to-many and many-to-one relationships (14). Such a construct could provide a functional architecture to a theory that demands “economical” solutions (i.e., to “do more, better or faster, with less”). As recently described, one-to-many/many-to-one designs can estimate both synergy and pleiotropy (11, 15).

To validate any method, the first step is to demonstrate construct validity. *Construct* is a concept that emerged in 1955 (16). It refers to make a judgment on something that cannot be measured directly. To explain this challenge, authors have mentioned the problem faced by disciplines other than Physics ‒which have physical standards, such as the one-meter-long bar made of platinum, adopted internationally after the 1875 Metre Convention (16)). Medicine in general and Immunology in particular lack such “standards”: there is no objective, universal and static standard for “health” or “immunity” (17).

Therefore, a new problem now affects infectious disease-related immunological methods: we need to measure concepts (always abstract, i.e., non-measurable), but, on a daily basis, we can only measure operations [consistently observable, i.e., measurable (17)]. For example, one “construct” could be that a particular cell type functions independently from the rest of the immune system. Consequently, measuring such cell type alone and in a non-structured format is sufficient to predict outcomes mediated by the immune system. An alternative construct could be that valuable information might result from structuring the data in ways that link two or more cell types so that multi-cellular interactions can be evaluated.

Because constructs may not function as expected, the assessment of construct validity is the priority of validation studies (16). They should be followed by examinations of *internal* validity, in which the influence or influences of other variables or “local” conditions on the construct are explored, such as co-morbidities. For instance, a construct might be considered valid when more or novel information is extracted from structured data than from non-structured data. However, if no extra information is extracted from structured data when co-morbidity is considered, then internal validity is not documented. Only after construct and internal validity have been documented, additional studies (conducted in other populations/places/times) could explore *external* validity, i.e., the influence of factors that exceed the host-pathogen interaction, such as the environment (18).

These considerations illustrate some of the numerous challenges that validations of immunological methods face. Other aspects to consider include: (i) differentiation between methods and techniques, (ii) discrimination between non-structured and structured information; (iii) implications of bottom-up vs. top-down approaches, (iv) selection of methods that foster discovery, invention, or both; and (v) differentiation between statistical significance and biomedical discrimination.

While techniques are not meant to answer scientific questions (they are just a means to an end), methods are theory-related and, in principle, can answer scientific questions (19). To that end, information science may be considered. While non-structured data may be non-informative, structured data may generate information that eventually produces knowledge which, after further translations, can support decisions ‒the Data-Information-Knowledge-Wisdom or DIKW pyramid (20).

Bottom-up and top-down approaches are expressions of the methodological approach adopted. Bottom-up approaches (referred to as upward causation) derive inferences from the analysis of primary (non-structured, “low-level”) data. In contrast, Complexity theory predicts that system-level information (i.e., highly structured data) can display “emergent” properties, which are not shown by low-level (non-structured) primary data. While reductionist methods ignore downward causation, non-reductionist approaches accept both downward and upward causation (21–23).

Emergent properties differ from resultant properties (7). While resultant properties can be predicted from the information provided by low-level, non-structured data, emergent properties cannot be predicted from or reduced to primary data. Methods that explore complex (system-level) emergent properties differ markedly from those that assume all individuals are similar when randomly selected (24).

Methods also differ in their consequences. Some methods allow doing something desirable but previously impossible to be conducted (“inventions”), while other methods (“discoveries”) identify something pre-existing but previously unknown (25, 26).

One example of an ‘invention that discovers’ is introducing complexity into the data (i.e., data structuring), followed by biological validation. As Brown and Botstein stated, ‘*the goal is to discover things we neither knew nor expected, and to see relationships and connections among the elements, whether previously suspected or not … this process is not driven by hypothesis and should be as model-independent as possible’* (27).

These considerations matter when the goal is not to rule in or out a hypothesis related to an average but to make, immediately, a medical decision. In Biomedicine, the priority is to separate what is different and bring together what is similar (28).

Biomedical discrimination can be facilitated when the properties of complex systems are considered, such as circularity, spatial relativity (ambiguity), and emergence (11). These properties are very well conserved in evolution: they are shown by human and non-human mammals as well as avian species (29–31). One consequence of biological complexity is that bottom-up approaches cannot anticipate outcomes associated with emergent properties –to that end, top-down methods are needed (32).

If, analyzing the same data, different methods vary in the information provided, the one eliciting *emergent* information (preexisting but previously unobserved) should be prioritized (23). New methods should also address the limitations of classic experimental designs (9). Because co-morbidities affect most people after 55 years of age and experimental animals are unreliable models for predicting human reactivity to many pathogens and drugs, trials that ignore co-morbidities or rely on studies conducted with inbred mice are likely to be invalid (10, 33). While randomized clinical trials may claim internal validity, they lack external validity (24). In contrast, pattern recognition-based designs that detect immune profiles previously unknown may result in substantial clinical efficacy, effectiveness, and validity (34–36).

These considerations are here addressed while investigating COVID-19 patients. Because it is prone to “discover”, a non-reductionist design that explicitly captures ‘one-to-many/many-to-one’ interactions was adopted (15). The chosen model also meets the requirements of personalized medicine and helps evaluate drugs and vaccinations (37). Approaches that satisfy such needs show *temporal directionality* (38). While tested for the first time in relation to COVID-19, the non-reductionist approach has been previously explored in hantavirus infections, sepsis, HIV, and other infections (11, 15).

This study aimed to evaluate whether a non-reductionist method can (i) extract more information than alternatives and prevent errors, such as confounding; (ii) predict outcomes, such as survival or non-survival to SARS-CoV-2 infection; and (iii) provide information that promotes personalized medicine. The central research question was: *does a method that estimates complexity inform the same as, more, or less than reductionist alternatives*?

## Materials And Methods

### Participants – Study I

A non-interventional, observational, and retrospective cohort study was based on hematological data collected from laboratory-confirmed COVID-19 individuals admitted to the Vardhman Mahavir Medical College (VMMC) and Safdarjung Hospital of New Delhi, India. In-hospital mortality within 90 days of hospitalization was recorded. Demographic, clinical, and outcome data were extracted from electronic medical records. Diagnoses and treatments for novel coronavirus pneumonia were made as described elsewhere (https://www.mohfw.gov.in). The investigated population was composed of 19 women and 32 men (51 patients), who contributed 98 temporal observations. Of those, 73 and 25 temporal observations corresponded to non-survivors and survivors, respectively. This study was conducted according to the protocol approved by the VMMC Institutional Ethics Committee (IEC/VMMC/SJH/Project/2020-08/CC-52).

### Participants – Study II

Following protocol ID:21-002778, CBCs collected from 101 COVID-19 patients (13 non-survivors, 88 survivors, who contributed 286 temporal observations) hospitalized at Mayo Clinic (Jacksonville, Florida, United States) were analyzed in reference to 30-day, in-hospital mortality.

### Inclusion and Exclusion Criteria

In both populations, all cases were treated at or before August 2020. Subjects older than 18 years of age, with SARS-CoV-2 positive test results conducted within 72 hours of admission, radiographic changes consistent with COVID, and deemed to be at risk of severe illness were enrolled. Discharge was based on negative testing and alleviation of life-threatening conditions. Subjects were excluded if they had a history of or were treated for immunosuppression, malignancy, pregnancy, or had been hospitalized for three or more weeks in the previous six months. **Supplementary Tables S1**–**S3** describe the demographic and clinical features of all participants.

### Study Data Collection

In population I, nasopharyngeal and oropharyngeal swabs and blood samples were conducted at and after admission. The total leukocyte count (TLC) and its differential percentages were performed with a hematology analyzer (Horiba). More than two dozen co-morbidities were investigated (**Supplementary Table S4**). In population II, similar procedures were conducted by Mayo Clinic diagnostic laboratories in accordance with established protocols (https://www.mayocliniclabs.com/florida/).

### Molecular Diagnosis

The diagnosis was based on detecting viral RNA in nasopharyngeal and/or oropharyngeal swabs using real-time reverse transcription-polymerase chain reaction [qRT-PCR (39)]. Swabs were collectively pooled in a viral transport medium (VTM; Himedia) followed by viral RNA extraction utilizing commercial kits [Qiagen (40)]. Using a thermal cycler (Bio-Rad, in population I; COBAS 8000 modular analyzer, in population II), a two-step strategy for the diagnosis of COVID-19 was followed as described elsewhere (41). The initial screening was targeted for the *E* (envelope) gene. Subjects positive in the screening test were confirmed by targeting a SARS-CoV-2 specific *RdRp* (RNA dependent RNA polymerase) gene and an ORF-1b-nsp14 gene (42, 43). Individuals yielding at least one positive result for either gene were regarded as infected with SARS-CoV-2 (44).

### Data Analysis

To capture biological complexity and, consequently, to detect emergent patterns, pattern recognition was pursued with a proprietary algorithm (US patent 10,429,389 B2) described elsewhere (15, 28). The algorithm creates dimensionless indices, which tend to reveal distinct data patterns. Such indices are temporary guides that lack biomedical meaning (here identified with letters in italics, e.g., *AAA*), whose only purpose is to reveal distinct patterns. Once such patterns are detected, the data are partitioned into subsets that include patients immunologically similar within but dissimilar across subsets. After the data are partitioned, the immunological content of all subsets is analyzed statistically. The biomedical significance of this method is determined by comparing its ability to separate patient subsets that differ in outcomes (survival vs. non-survival) with that of non-structured data (the classic CBC). One variation of this approach is to generate three- or four-dimensional (3D/4D) data structures that meet two criteria: (i) elimination of data variability (noise) from all dimensions but one, and (ii) detection of temporal changes even when consecutive observations are conducted within a short period and only a single patient is under evaluation. A structure that exhibited one data point-wide line of observations (*1dpwlo*) was chosen because it seems appropriate for the needs of personalized medicine (15).

Principal Component Analysis and χ^2^ tests were conducted using *Minitab 19* (Minitab Inc, State College, PA, USA). The same software package was used to produce graphics.

## Results

### The Classic Method

**Figure 1:**
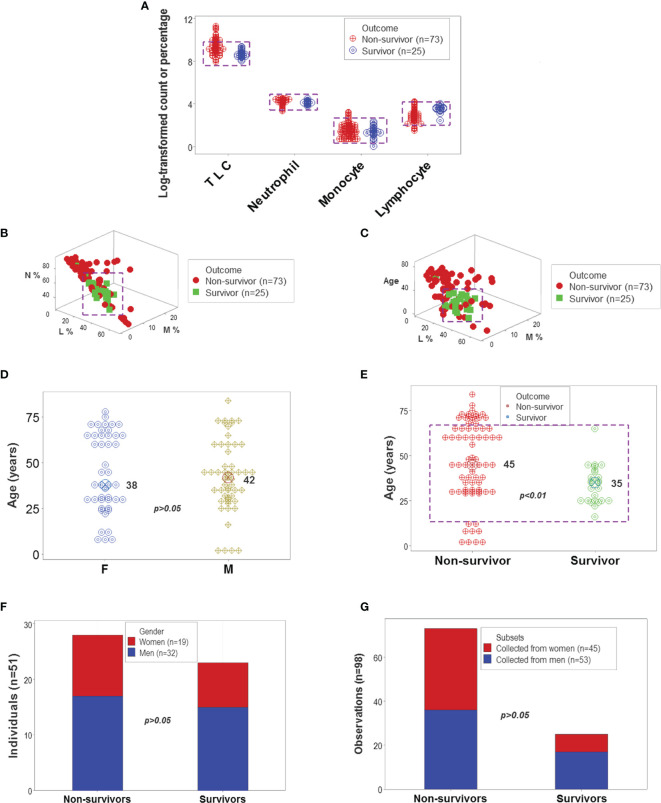
Leukocyte-demographic summary of the New Delhi population. Survivor- and nonsurvivor-related overlapping observations were observed when the total leukocyte counts (TLC) and relative percentages of blood neutrophils (N), monocytes (M) or lymphocytes (L) were analyzed (rectangles, **(A)**. Three-dimensional (3D) analysis of the data did not remove data overlapping even after age was considered (rectangles, **(B, C)**. Age did not differ significantly between female and male participants **(D)**. While the median age was significantly lower in survivors than non-survivors, most observations of both outcomes displayed overlapping values **(E)**. Lack of statistically significant differences between the gender of participants and disease outcomes were further demonstrated when the unit of data analysis was the individual (n=51 observations) and also when all 98 temporal data points were considered **(F, G)**.

**Figure 2:**
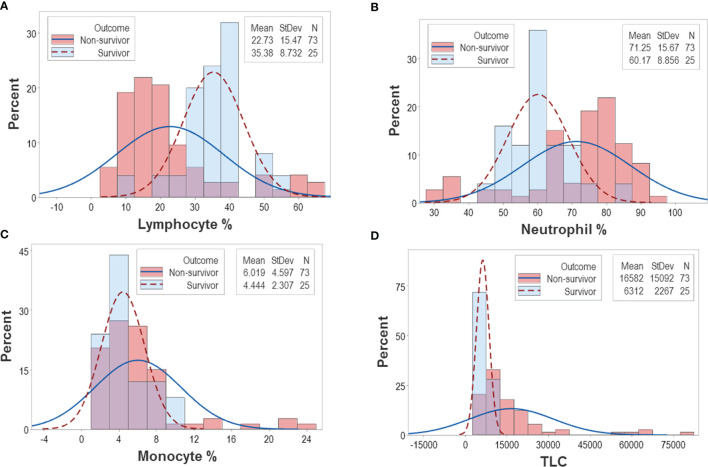
Continuous distributions of New Delhi leukocyte data. Overlapping data distributions were also observed when data points ‒which, inherently, are discrete or discontinuous‒ were assumed to be continuous **(A–D)**. Considering that the highest value of each line represents the cutoff that separates survivors from non-survivors and projecting these lines over a histogram, non-survivor observations are depicted as dark pink bars and survivor observations are displayed as sky blue bars. Assuming that survivors are “positive” results and non-survivors are “negative” results, purple bars display the magnitude of false-negative and false-positive results, i.e., survivors that show observations within the non-survivor side of the plot (“false-positives”) and non-survivors that show observations within the survivor side of the plot (“false-negatives”). It is shown that misclassifications (purple bars) cover a substantial if not the whole range of the data.

The analysis of CBC counts, or percentages did not distinguish outcomes. Overlapping data distributions of the total leukocyte count (TLC) and the percentages of neutrophils (N), lymphocytes (L), or monocytes (M) prevented to differentiate survivors from non-survivors (**Figure 1A**). Discrimination did not improve when 3D relationships and age were considered (**Figures 1B, C**). While age was marginally higher in males and more males than females were investigated, neither age nor gender explained outcomes (**Figures 1D–G**). Whether expressed as the total number of patients or the total number of observations, the proportions of gender-related cases did not differ statistically (*p*>0.05, Chi-square test, **Figures 1F, G**). While the median age differed statistically when outcomes were analyzed (*p*<0.01, Mann-Whitney test), substantial data overlapping exhibited by survivors and non-survivors prevented their differentiation (rectangle, **Figure 1D**). Data overlapping remained even when the optimal cutoff was selected –the highest point of the curve indicating the data distribution of each outcome class. As graphically indicated by purple bars, numerous survivors and non-survivors were observed on both sides of the cutoff (**Figures 2A–D**).

### The Pattern Recognition-Oriented Method

**Figure 3:**
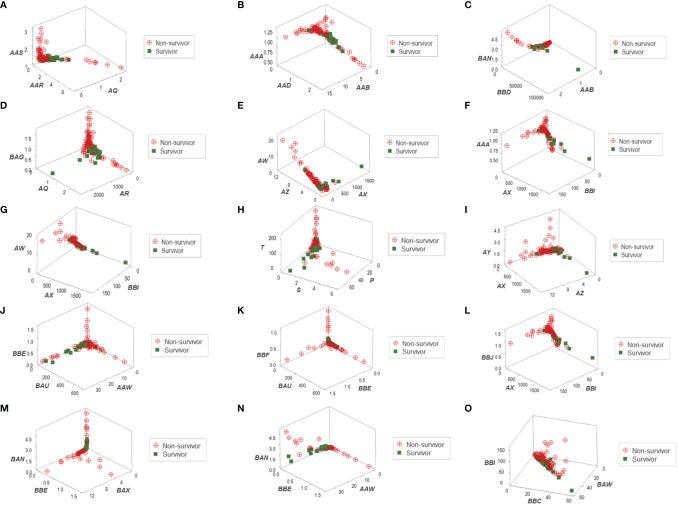
Three-dimensional pattern recognition of the New Delhi leukocyte data. Fifteen 3D data structures derived from the blood leukocyte data were explored for the presence of distinct (non-random) patterns. Each axis of each plot describes hypothetical and dimensionless indicators designed to express multi-cellular relationships, which are identified with two or three letters in italics **(A–O)**. By reporting outcomes, this construct can simultaneously (i) show distinct patterns, if they exist (e.g., a perpendicular data inflection) and (ii) reveal whether one (or both) outcome(s) is/are clustered. To prevent artifacts, this process depends on redundancy: inferences are based on, at least, two separate data structures. This set of figures includes data structures showing: (i) a single (and perpendicular) data inflection **(A)**; (ii) a data bifurcation **(B)**; (iii) a perpendicular data inflection with some survivors clustered in one data segment **(C)**; (iv) a rendundant expression **(D)**; (v) a perpendicular data inflection with most survivors clustered in one data segment **(E)**; (vi) a data bifurcation with a cluster that includes most survivors **(F)**; (vii) a perpendicular data inflection that includes a data segment only composed of survivors **(G)**; (viii-xi) four structures that reveal three data segments, perpendicular to one another **(H–K)**; (xii) a partially redundant structure (spatially similar to B), which shows a cluster of survivors **(L)**; (xiii) three perpendicular data inflections that include two data segments only composed of non-survivors **(M)**; (xiv) a partially redundant structure (spatially similar to G), which differs in two aspects: it identifies a data segment only composed by non-survivors, which is perpendicular to the remaining observations **(N)**; and (xv) a partially redundant structure (similar to N) which provides an additional indicator that separates non-survivors (high values of the verticql axis) from survivors and displays very low values in the vertical axis **(O)**.

**Figure 4:**
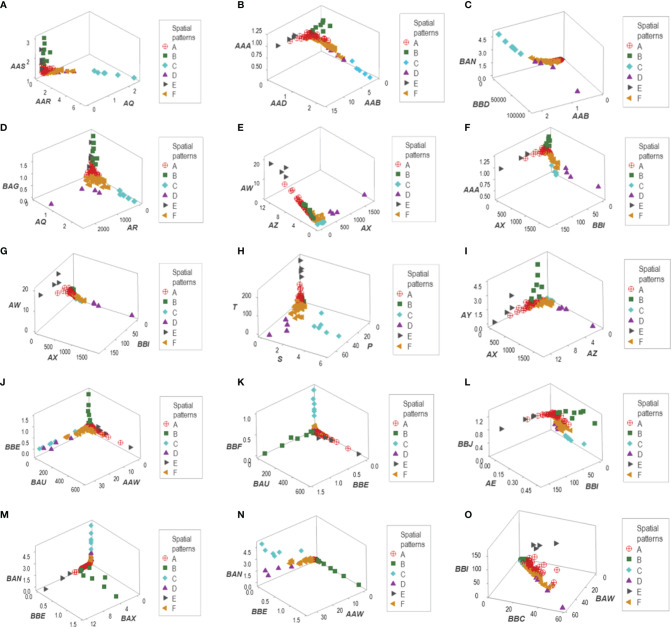
Data partitioning (labeling) of the New Delhi leukocyte data. After a substantial number of distinct patterns was observed (**Figure 3**), the patient identity of each data segment was identified. When at least two data structures identified the same group of patients, each patient group is identified with a unique identifier. This process identified six data groups (identified as ‘A, B,…F’). For instance, group ‘C’ included observations that were easily identified: they were a separate (non-overlapping) cluster, recognized by, at least, four data structures **(A–D)**. Two other patient groups (‘D’ and ‘E’) were also identified by the spatial patterns shown by five data structures **(E–I)**. A third patient group (‘B’) was unambiguously detected by five data structures **(J–N)**. The two remaining patient groups (‘A’ and ‘F’) were differentiated by a double process: (i) from one another, they were distinguished by a perpendicular data inflection **(H, J)** and (ii) from the remaining patient groups, by default. Patient group ‘E’ was also identified by the data structure **(O)**.

Distinct data patterns (such as perpendicular data inflections) supported the view that outcomes were not randomly distributed (**Figures 3A–O**). Such an inference was further supported when redundant data patterns differentiated six subsets of patients (**Figures 4A–O** and **Supplementary Table S2**).

**Figure 5:**
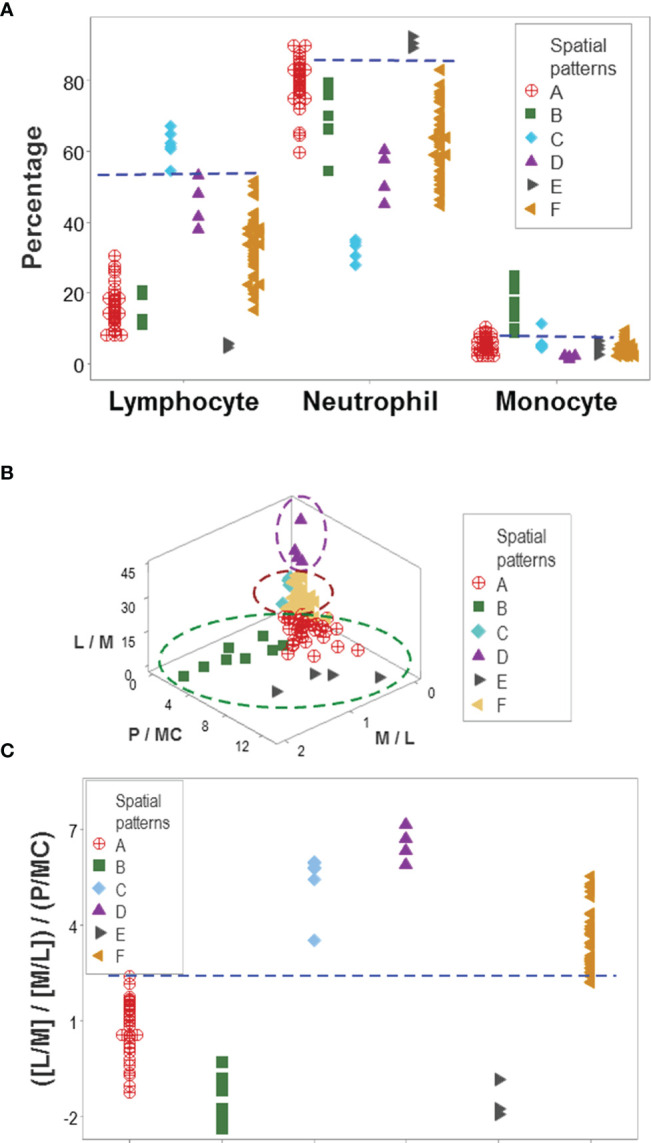
Immunological content of patient groups. Non-overlapping percentages of lymphocytes and neutrophils distinguished two data groups ('C' and 'E') from all the remaining patient groups, while non-overlapping intervals of at least one cell type differentiated group ‘B’ from four of the five remaining groups **(A)**. A complex ratio that captured five multi-cellular relationships (L/M, M/L, [L/M/M/L], P/MC, and [[L/M/M/L]/P/MC]) differentiated, with non-overlapping data intervals, patient groups ‘A’, ‘D’, and ‘F’ from one another **(B)**. Discrimination was not due to any one (single or complex) variable but to interactions: when the three constitutive elements displayed in **(B)** were analyzed individually (the L/M, M/L and P/MC ratios), confounding was observed: five of the six immune profiles were mixed. This means that the emergent information that discriminates only occurs when the most complex (system-level) interaction is assembled in 3D space **(C)**. L, lymphocytes; N, neutrophils; M, monocytes; P, phagocytes (N and M); MC, mononuclear cells (L and M).

While leukocyte data did not discriminate when each cell type was analyzed in isolation (**Figures 2A–C**), the same data showed total or quasi-total non-overlapping data intervals when immune profile-related patterns were considered (**Figure 5A**). Discrimination was not the result of any one spatial analysis but a process that included many data combinations and spatial-temporal assessments that could include many perspectives. **Figures 5B, C** supported the hypothesis that discrimination cannot be achieved with pre-established indicators: when three biologically interpretable indices were investigated in 3D space (the lymphocyte/monocyte [L/M], the monocyte/lymphocyte [M/L], and the phagocyte/mononuclear cell [P/MC] ratios), five of the six patient subsets overlapped (**Figure 5B**). Yet, when the same indices were included in one complex ratio, patients were separated into two non-overlapping groups (**Figure 5C**).

### Applications in the Evaluation of Disease-Related Hypotheses

**Figure 6:**
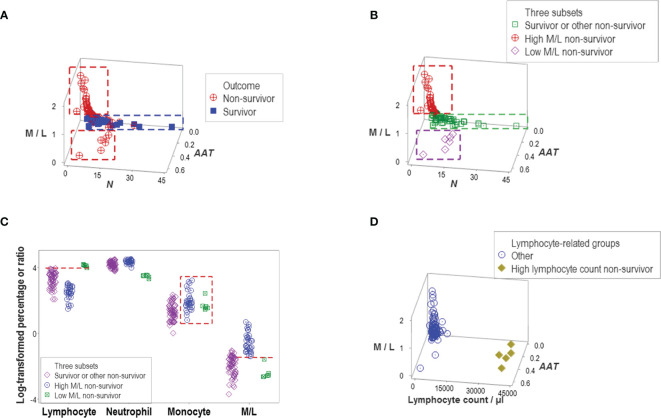
Evaluation of hypotheses and discovery (I). A subset of non-survivors showed very low values of the M/L ratio **(A)**. This finding seemed to disprove the hypothesis that only high M/L values are associated with disease severity. Instead, at least two subtypes of non-survivors were discovered, which displayed high and low M/L values, respectively. Because another data subset included other non-survivors and all survivors, three subtypes of non-survivors were found **(B)**. The monocyte percentage did not distinguish high from low M/L non-survivors (box, **(C)**. Discrimination of these two subtypes of non-survivors was due to a lower percentage of lymphocytes, which are observed in the high M/L groups (horizontal lines, **C**). The hypothesis that lymphopenia is always associated with disease severity was not supported: five non-survivors did not show lymphopenia **(D)**.

Indicators that expressed distinct patterns helped evaluate earlier claims on COVID-19, such as the double hypothesis that postulates COVID-19 disease severity is associated with an increased M/L ratio and lymphopenia. It was shown that increased values of the M/L ratio do not always characterize non-survival –a subset of non-survivors revealed very low values of the same ratio (**Figure 6A**). Survivors and other non-survivors were included in the remaining group (**Figure 6B**). Monocyte percentages did not predict M/L ratios (**Figure 6C**). In contrast, high lymphocyte values (even higher than those shown by the group that included all survivors) were noticed in the low M/L non-survivor group (**Figure 6C**). The hypothesis that lymphopenia always predicts disease severity (i.e., death) was deemed invalid: five non-survivors did not display lymphopenia (**Figure 6D**).

**Figure 7:**
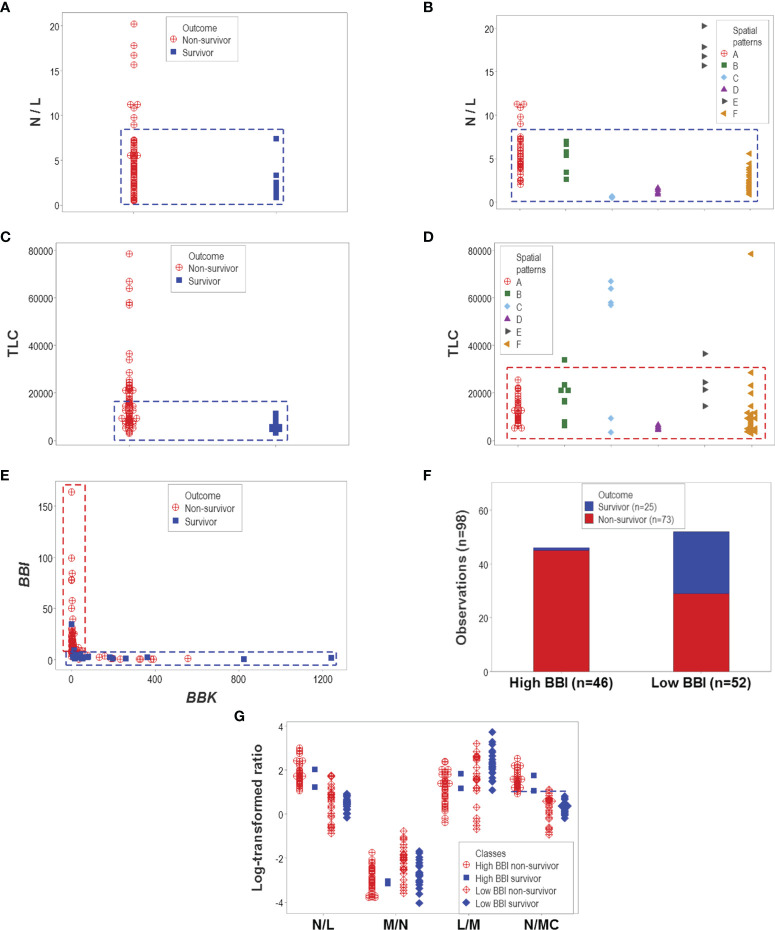
Evaluation of hypotheses and discovery (II). The hypotheses that claim high values of the neutrophil/lymphocyte (N/L) ratio or the total leukocycte count (TLC) are associated with disease severity were also tested. Neither hypothesis was supported: both the N/L ratio and the TLC confounded different outcomes and immune profile-defined patient groups **(A–D)**. However, when two dimensionless indicators (named ‘BBI’ and ‘BBK’) were explored, a one data point-wide line of observations (1dpwlo) exhibited a perpendicular inflection that distinguished two data subsets **(E)**. One of the data subsets (named ‘high BBI’) was predominantly (97.8% or 45/46) composed by non-survivors (*p*<0.01, χ2 test, **(F)**. Most high BBI non-survivors displayed a higher ratio of neutrophils over mononuclear cells (N/MC ratio) than most non-survivors **(G)**. Therefore, the analysis of complex but hypothetical immunological relationships discovered a prognosticator: high values of the N/MC ratio may predict non-survival.

Other indicators reported to be associated with disease severity also failed to discriminate. Both the neutrophil/lymphocyte (N/L) ratio and the total leukocyte count (TLC) confounded different outcomes (**Figures 7A–D**). However, one particular data structure (a one data point-wide line of observations or *1dpwlo*) exhibited an orthogonal pattern that distinguished two data subsets (here named high or low *BBI*, **Figure 7E**). One of the data subsets was predominantly (97.8% or 45/46) composed of non-survivors (high *BBI* subset, *p*<0.01, χ^2^ test, **Figure 7F**). The ratio between neutrophils and mononuclear cells (N/MC) was higher in the group predominantly composed of non-survivors (high *BBI* group), which only marginally (7.7% or 4/52) overlapped with the low *BBI* group of non-survivors (**Figure 7G**).

### Prognostic Applications

**Figure 8:**
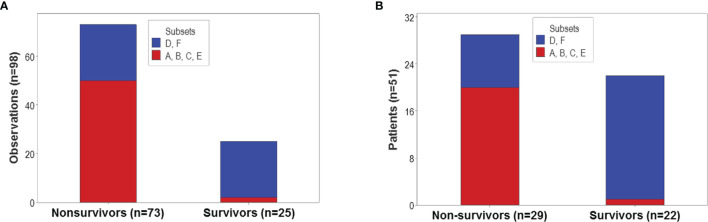
Population-level prognosis. The proportion of survivor-related observations differed statistically among the six immunological groups (*p*<0.01, χ2 test). Most (92% or 23 out of 25) survivor-related observations were clustered into the ‘D’ or ‘F’ patient groups; in contrast, 68.5% (50 out of 73) nonsurvivor-related observations were found within the remaining four groups **(A)**. Similar proportions were observed when patients –not observations− were the unit of analysis: 95.5% (21 out of 22) survivors were classified as either ‘D’ or ‘F’ patients, while 68.9% (20 out of 29) non-survivors were clustered within the remaining groups **(B)**.

The proportion of survivors differed statistically among the six patient groups (*p*<0.01, χ2 test): 92% (23/25) of all observations collected from survivors were clustered into the ‘D’ or ‘F’ groups (**Figure 8A**). In contrast, less than one third of all non-survivors were classified into the ‘D’ or ‘F’ groups. Similar proportions were observed when patients –not observations− were the unit of analysis: 95.5% (21/22) of all survivors were either ‘D’ or ‘F’ patients (**Figure 8B**).

**Figure 9:**
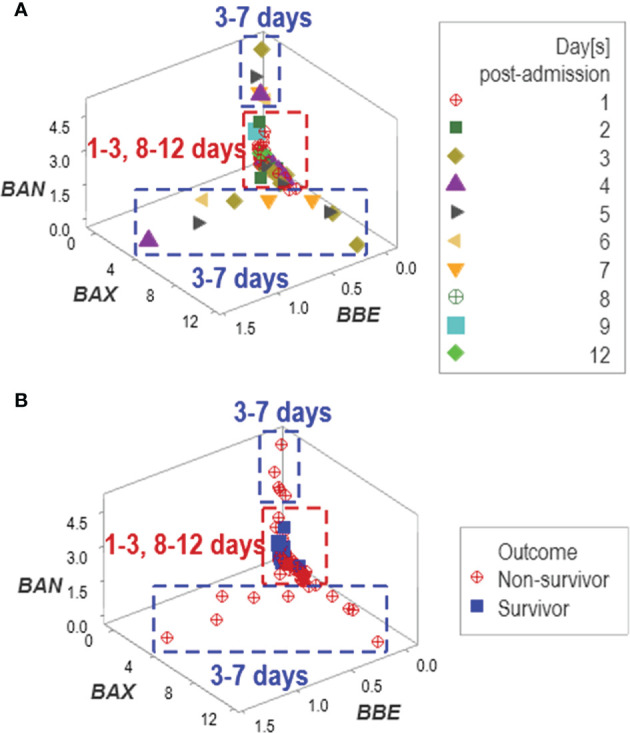
Assessment of potential errors patterns and additional discovery The data collected in the New Delhi study exhibited circular patterns. Data circularity was deduced because: (i) the same data cluster included observations from two periods (between 1 and 3, and 8 and 12 days post-admission), and (ii) two clusters only included data reported between 3 and 7 days **(A)**. While such expressions might suggest ambiguity ‒and, consequently, lack of discrimination‒, pattern recognition detected actionable information: two of the three clusters only included non-survivors **(B)**.

In addition to *emergence*, other properties typical of complex systems were observed, such as data *circularity*. At least two temporal loops of data circularity were deduced because one data cluster included observations from two periods (between 1 and 3, and between 8 and 12 days post-admission), while two additional clusters only included data reported between 3 and 7 days (**Figure 9A**). Data circularity was not a random event: two clusters only included observations generated by non-surviving patients (**Figure 9B**).

**Figure 10:**
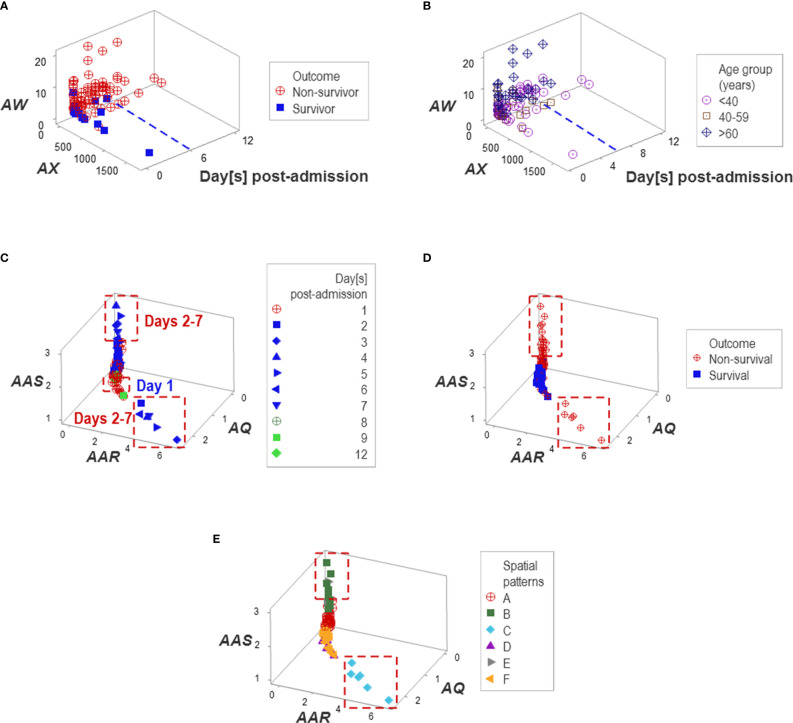
Temporal patterns. No survivor was reported in the Indian population after six in-hospital days **(A, B)**. Day-1 post-admission observations differed from later observations: two subsets of later observations were detected, which only involved non-survivors **(C, D)**. Patient groups were predominantly explained by temporal patterns, e.g., one pattern was only explained by group ‘C’ and a second pattern was mainly explained by group ‘B’ **(E)**.

Additional temporal patterns were observed: regardless of age, non-survivors remained hospitalized six days or less (**Figures 10A, B**). Corroborating data circularity, day-1 post-admission observations differed from later (day 2-7) observations. Two subsets of later observations were detected, which only included non-survivors (**Figures 10C, D**). These two groups of non-survivors ‒predicted as such between 2 and 7 days post-admission‒ were identified as groups ‘B’ or ‘C’ (**Figure 10E**).

**Figure 11:**
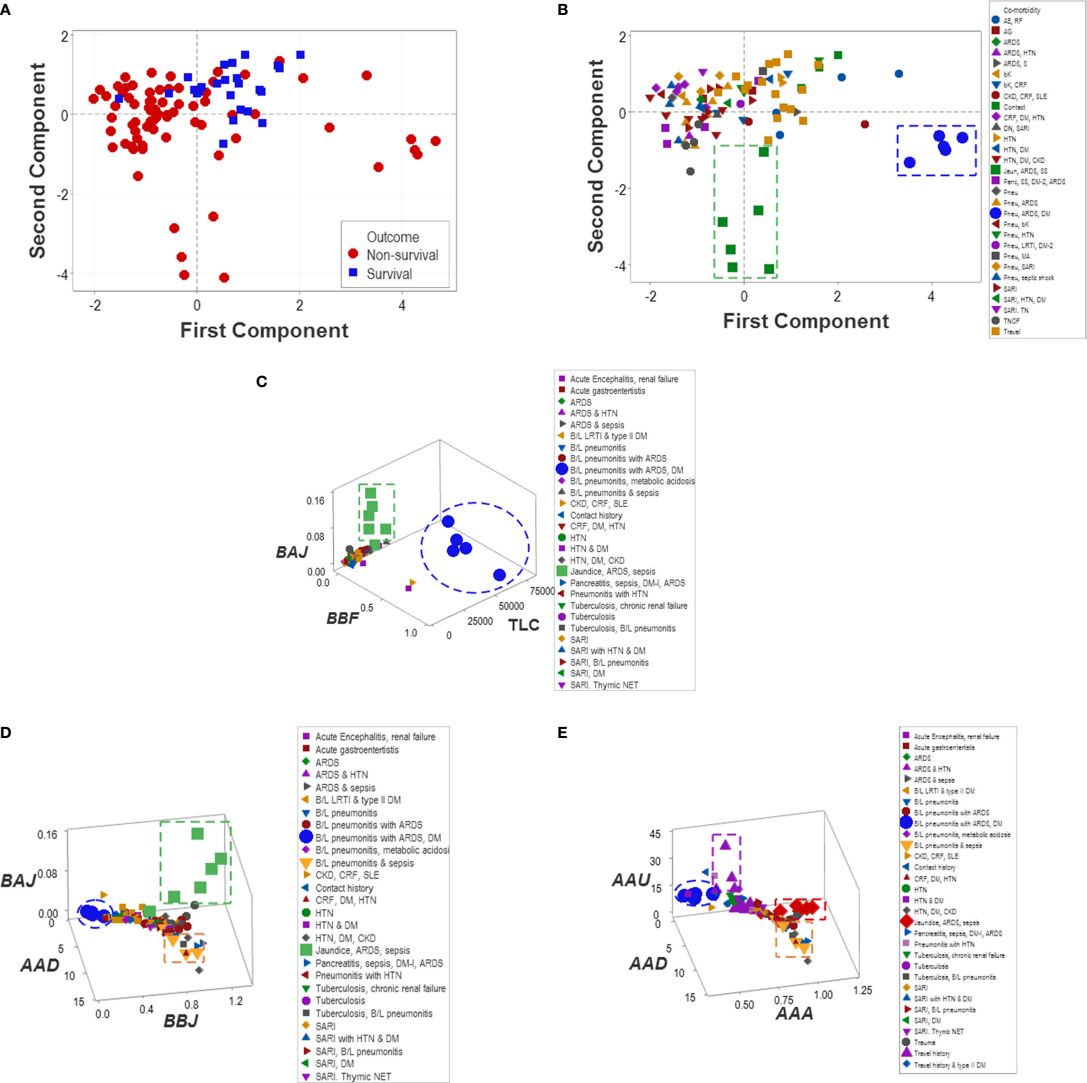
Reductionist and non-reductionist analysis of co-morbidities. A method meant to reduce dimensions (Principal Component Analysis or PCA) was applied to explore outcomes and co-morbidities. The PCA did not distinguish survivors from non-survivors **(A)**. While the PCA discriminated two sets of co-morbidities (namely, (i) pneumonitis (B/L pneu), acute renal distress syndrome (ARDS) and septic shock (SS) (green squares) as well as (ii) B/L pneu, ARDS and diabetes mellitus type 1 (DM) (blue circles, **B**), such sets were also detected by the non-reductionist method, which, in addition, differentiated (iii) travel history (purple triangles) and (iv) two subsets of sepsis (red diamonds and yellow triangles, **C–E**).

Unlike multi-cellular indicators, neither isolated variables (such as the TLC and the percentages of lymphocytes, neutrophils, and monocytes) nor dimension-reducing approaches (Principal Component Analysis or PCA) separated non-survivors from survivors (**Figure 11A**). While the PCA only distinguished two groups of co-morbidities (**Figure 11B**), the non-reductionist method identified four sets of co-morbidities (**Figures 11C–E**).

### Applications in Personalized Medicine

**Figure 12:**
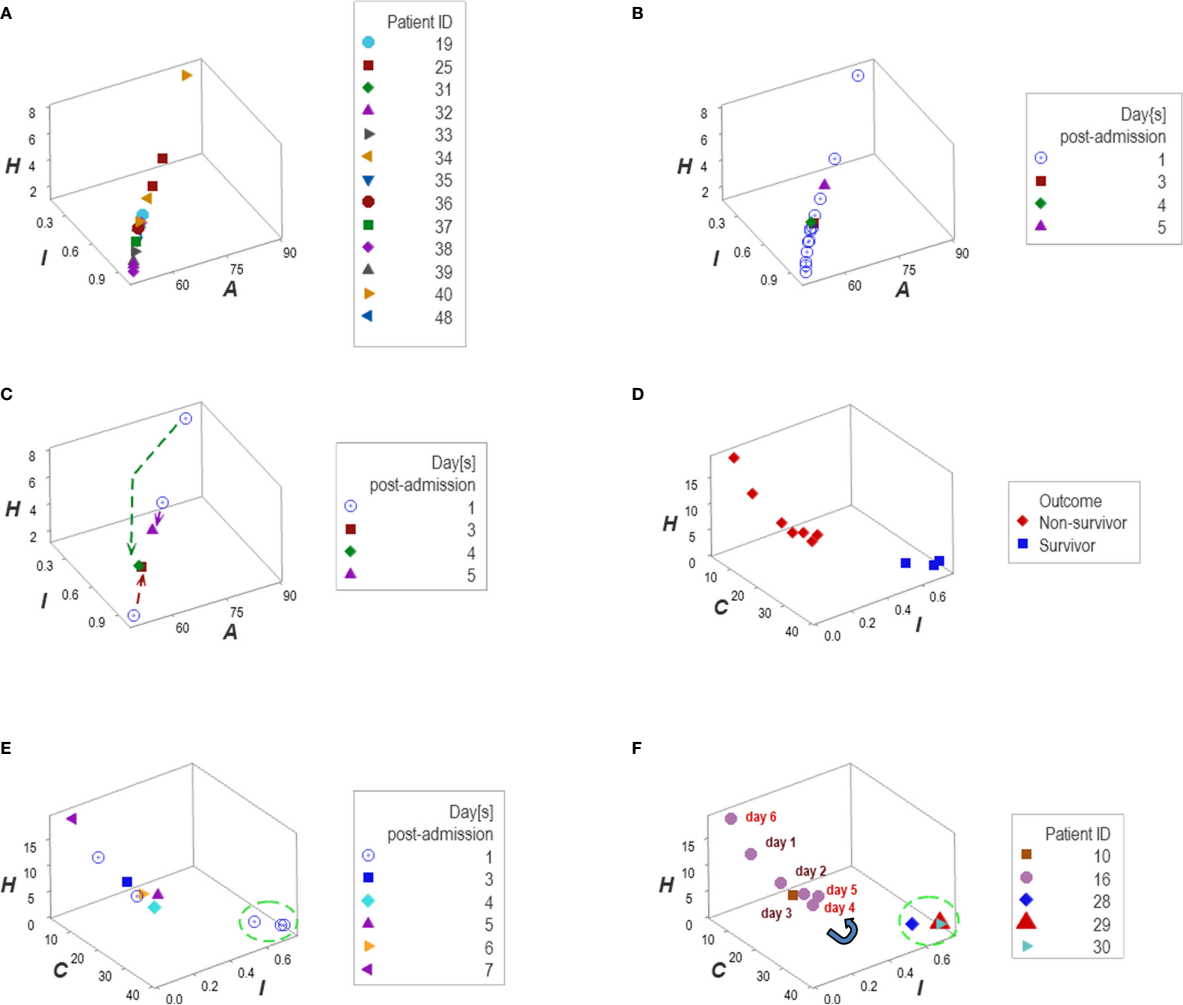
Personalized, directionality-based prognostics. Data structures designed to remove data variability from all dimensions except one facilitated personalized assessments **(A–F)**. For example, the data of 13 patients that reported a travel history displayed a one data point-wide line of observations (1dpwlo, **(A, B)**. This data structure removes variability from all dimensions except one (along the line). Consequently, temporal changes can only occur along the line, and they will be detected even with a single observation (inferences are based on the directionality shown by arrows, not numerical values). Panel **(C)** shows the temporal data patterns generated by three patients, which expressed both a top-down flow (two individuals) and a bottom-up temporal directionality (one individual, **(C)**. Applications of temporal 1dpwlo are depicted in panels **(D, E)** they describe one 1dpwlo with different outcomes clustered at each end of the line of data **(D)**. When time is considered, non-survival is predicted when, over time, observations move from the right to the left **(D, E)**. Therefore, a single change in temporal data directionality (an arrow that changes directions) is sufficient to predict, at a personalized level. For instance, patient #16 was showing a left-to-right, top-down temporal flow between day 1 and 4 (a survival prediction, panel **(F)**. However, by day 5 the directionality of the data reversed, which predicted non-survival **(F)**. Panel **(D)** confirms such a prediction.

Personalized analyses were facilitated by one data point-wide line of observations (*1dpwlo*). These data structures were designed to remove data variability from all dimensions except one (along the line) and express temporal data directionality (arrows that indicate where the data came from). These structures distinguished patients with a travel history and those who experienced hypertension (**Figures 12A–F**).

### Reproducibility and Statistical Validity

External validity (generalizability) was explored together with statistical validity. To that end, a similar research design was applied to a group of 101 COVID-19 patients treated in Jacksonville, Florida, United States. Because several CBCs were collected from each patient, 286 temporal observations were available for analysis.

**Figure 13:**
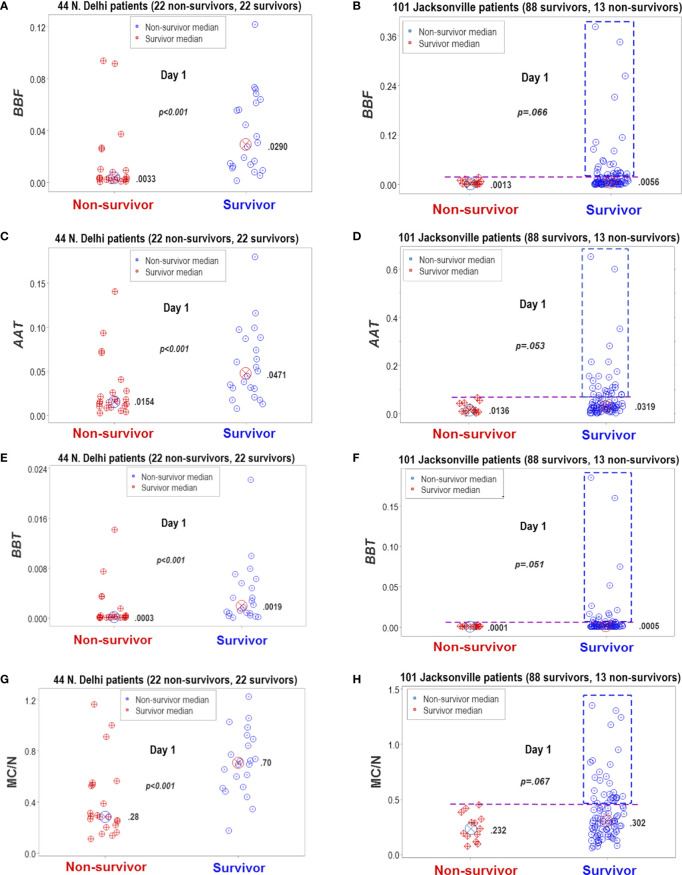
Statistical significance and biomedical discrimination across populations. Some complex and dimensionless indicators (named *BBF*, *AAT*, and *BBT*) were explored at the first hospitalization day, both in the Indian dataset and in data collected from patients treated in Jacksonville, Florida, United States **(A–F)**. To validate these indicators, the mononuclear cell/neutrophil ratio was used **(G, H)**. Supporting the hypothesis that the dimensionless indicators were biologically valid, both populations reported similar findings: the dimensionless indicators and the M/CN ratio approached statistical significance when survivors and non-survivors were compared or were statistically significantly higher in survivors than in non-survivors. While differences between non-survivors and survivors reached statistical significance in the New Delhi population, they displayed overlapping data distributions that did not facilitate discrimination. In contrast, the Jacksonville population approached (but did not reach) statistical significance and many survivors displayed a substantial number of observations clearly above the upper limit of non-survivors (rectangles, **(B, D, F, H)**. Consequently, two inferences were supported by the data: (1) the non-reductionist method appears to possess external validity (it is robust to population-related variability), and (2) statistical significance is not synonymous with biomedical discrimination ‒one may occur without the other.

**Figure 14:**
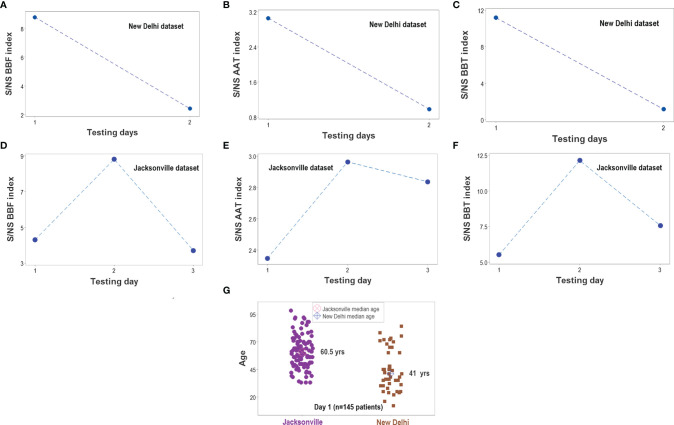
Survivor- and nonsurvivor-related temporal immunological trends. When time was considered, the three complex indicators reported in **Figure 13** displayed similar magnitudes in both populations **(A–F)**. The ratio between survivors (S) and non-survivors (NS) was higher than 1 at all time points. Immunological differences between survivors and non-survivors were not explained by demographic factors: the median age was much lower in the New Delhi than in the Jacksonville group **(G)**. Note I: given the few temporal data points available in the Indian dataset, all observations collected at day 2 or later were merged; i.e., day-2 values for the Jacksonville dataset do not necessarily correspond to day-2 values of the New Delhi dataset. Note II: while a total of 152 patients were investigated (51 from New Delhi and 101 from Jacksonville), day 1 observations only included 145 of such individuals. The difference is due to 6 N. Delhi patients whose first test was not conducted on hospitalization day 1.

The analyses demonstrated that, in both populations, biological discrimination might occur without statistical significance and vice versa (**Figures 13A–H**). Temporal trends also revealed similarities between the two populations (**Figures 14A–F**). Yet, demographic features differed between these populations: the median age of New Delhi patients was 19 years younger than the median age of Jacksonville patients (*p*<0.01, Mann-Whitney test, **Figure 14G**).

Recognition of data patterns facilitated applications. For example, in the New Delhi dataset, 72.7% (16/22) of all survivors were identified, at day 1, outside the cluster where non-survivors predominated (**Supplementary Figure S1A**). In the Jacksonville population. the same indicators discriminated, at hospitalization day 1, 36.4% (32/88) of all survivors (**Supplementary Figure S1B**).

When a different data structure was considered, 37.5% (34/88) of all Jacksonville survivors were identified at day 1 and, in addition, two subsets of survivors (‘A’ and ‘B’) were distinguished, which displayed non-overlapping distributions of the MC/N ratio (**Supplementary Figure S1C, D**). Unlike the New Delhi group (where the values of 5 of 22 non-survivors overlapped with survivors), the identification of Jacksonville survivors was 100% sensitive: no observation collected from a non-survivor was found within survivor subsets ‘A’ and ‘B’ (**Supplementary Figure S1C**). Also, 16 additional non-survivors were detected in the Jacksonville dataset when a third data structure was measured. Therefore, 54.5% (48/88) of all survivors were distinguished when three data structures were considered (**Supplementary Figure S1E**). With a fourth data structure, 5 more survivors were detected in the Jacksonville group, resulting in a cumulative detection of 60.2% (53/88) of all survivors being identified at day 1 (**Supplementary Figure S1F**).

Other potential applications included the detection of subsets of a given outcome, which may be found in different proportions across populations. For instance, in the New Delhi population, 68% (15/22) of all non-survivor observations were located within a distinct data subset, in which non-survivors represented 93.8 (15/16) of all data points. In contrast, only 23% (5/22) of all Jacksonville non-survivor observations were found in the same data range (**Supplementary Figures S1 G, H**).

**Figure 15:**
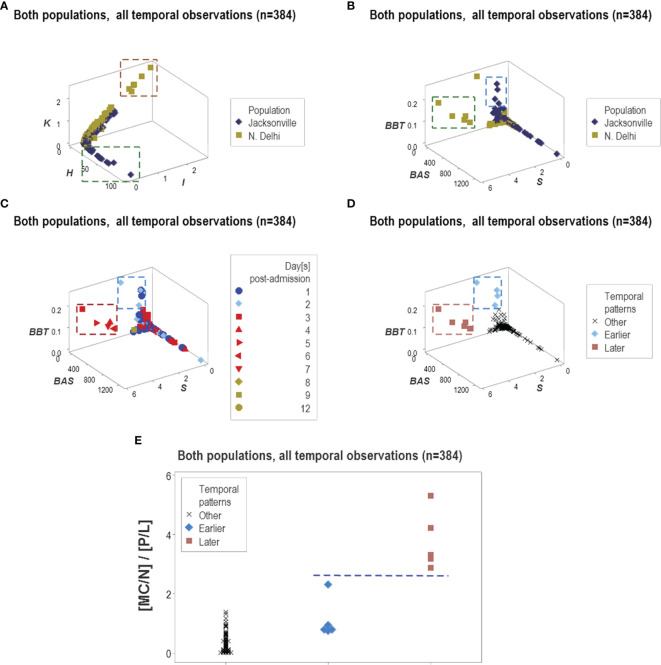
Temporal and population-specific patterns. Supporting both the generalizability and the informative potential of the non-reductionist approach, both similar and different inferences were found across populations **(A, B)**. Integration of immuno-pathology with pattern recognition and temporal assessments was also documented. For instance, two distinct data clusters observed when chronological data (hospitalization days) were observed **(C)** could be postulated to represent early or late inflammation **(D)**. The presumptive inflammatory phase was biologically supported when a biologically explicit (although complex) ratio was analyzed: early inflammatory processes are consistent with increased phagocyte/lymphocyte ratios and late or recovery processes tend to be characterized by higher mononuclear cell/neutrophil ratios, that is, lower values of the [MC/N]/[P/L] complex ratio are expected in early inflammation and higher values of the same indicator may be found in later stages **(E)**.

Because some data patterns were shared by both populations and some patterns were only displayed by one population, this methodology showed to be both robust and discriminant (**Figures 15A, B**). This approach also estimated temporal stages of the disease progression process. As depicted in **Figures 15C, D**, tentative temporal labels (early vs. late inflammation) can be generated by integrating chronological information (days after admission) with spatial data patterns. These tentative labels were consistent with the immuno-pathological literature: the double ([MC/N]/[P/L]) ratio is expected to be higher in the later or resolution phase of non-complicated inflammations than in earlier stages (**Figure 15E**).

## Discussion

### Overview

This study conducted the first evaluation of a non-reductionist methodology that explores system-level and dynamic properties of biological complexity in COVID-19 patients. While typical proofs-of-concept are limited to construct validity, this report explored the four fundamental threats to cognitive inferences: (i) construct, (ii) internal, (iii) external (generalizability or reproducibility), and (iv) statistical validity.

To estimate construct validity (16), CBC data helped compare the informative ability of the novel and the classic approach. The inclusion of patients with various co-morbidities investigated internal validity (18). The assessment of two populations affected by SARS-CoV-2 explored external validity (18). Statistical validity was estimated by considering both biomedical discrimination and statistical significance (28).

The reason for such a comprehensive analysis was the combinatorial and multi-level nature of the method investigated. An approach potentially influenced by many biological factors may require simultaneous investigations that involve numerous perspectives ‒not consecutive explorations in which only a few aspects interact.

### Methodological Rigor and Implications

In addition to several types of validity, methodological rigor was emphasized. While no consensus exists on the meaning of methodological rigor; some authors suggest that rigor reflects the reasoning employed behind the design, i.e., a process that includes grounds (empirical data), claims (theoretical conclusions derived from the data), and warrants, i.e., rules of reasoning applied to the data to make inferences (45). Accordingly, rigor was here conceptualized by nine domains that were categorized into three groups:

#### A. Data That Support and/or Discriminate

(i) individual-related (personalized) inferences,
(ii) population-related inferences,
(iii) time-related inferences (e.g., those that may distinguish early vs. late inflammation),
(iv) prognostic inferences (e.g., those that may distinguish late inflammation that leads to recovery from late inflammation that leads to chronicity),
(v) inferences on the validity of classic methodological concepts, including statistical significance and biomedical discrimination; and
(vi) subtypes within the same outcome (e.g., two or more types of survivors, as defined by immune profiles);

#### B. Designs of Data Structures That Estimate or Prevent

(vii) complexity (e.g., two or more levels of complexity, such as complex and straightforward ratios),
(viii) omissions (e.g., two or more data structures or redundant analysis), and/or
(ix) waste of time and other resources; and

#### C. Data Reports That Promote

(x) reproducibility of critical results by independent investigators and/or clinical applications.

This strategy translated as 103 plots described in 16 composite figures. While this emphasis on methodological rigor is uncommon in the literature, it is suggested it was necessary because this study is the first one that, within the context of COVID-19, explores not only a method but also a theory that considers the complexity of multi-level/multi-cellular and dynamic immunological interactions.

By exploring several co-morbidities reported in populations located in different continents, the analysis of complex and dynamic multi-cellular interactions revealed a language both flexible and robust, which could express both similarities and differences, as **Figures 15A, B** demonstrate. While binary methods are self-limiting (46), the one evaluated was not: it demonstrated it could distinguish three or more patient-related patterns.

While other technologies were not considered because this study prioritized rapid turnaround time and operational simplicity, the pattern recognition-oriented method was compatible with other technologies. For instance, when flow cytometry is used, a sub-cellular/cellular/supra-cellular integrated analysis is generated (47, 48).

It is suggested that the combinatorial nature of a method designed to capture both one-to-many and many-to-one relationships may lead to a new type of research publications. A single or few (reductionist) question(s) may be replaced by a large number of visualizations that, without pre-established cutoffs, attempt to uncover distinct data patterns that, immediately, can be biologically validated and converted into research and/or clinically actionable knowledge (20, 49).

### Major Findings

Despite demographic differences among patients, the analysis of complex multi-cellular indicators seemed applicable across populations. Specifically, the data supported ten inferences:

(i) *Data structuring* (e.g., creation of new metrics that capture multi-cellular interactions) is essential ‒the same data, non-structured, may lack meaning;
(ii) *Biomedical discrimination* (non-overlapping data distributions of biologically distinct outcomes, e.g., survivors and survivors) may differ from statistical significance;
(iii) Biologically grounded prognosis may be generated *early*,
(iv) Pattern recognition fosters *non-binary detection* (three or more entities, including two or more types of survivors and non-survivors, can be discriminated);
(v) *Error prevention*: emergent properties (e.g., data circularity) may reveal clusters that ameliorate *spatial relativity* (data ambiguity);
(vi) *Disease stage* (inflammatory phase) may be estimated in real time;
(vii) Without population-related averages, one data point-wide lines of temporal observations promote *personalized* information;
(viii) Data patterns can identify *departures from generic assumptions* (e.g., “lymphopenia is a hallmark of disease severity”) and discover *actionable information*;
(ix) *Rapid* and *translational* (clinician-friendly) information is generated, which does not require novel specialized training or time-consuming technology; and
(x) *Population-specific* information may also be identified.

Many, if not all, of these findings relate to a major construct used in Biomedicine since 1947: the “contingency or 2 x 2 table” paradigm (50, 51). This model lacks validity and promotes confounding. Because it is inherently binary (it only accepts two alternatives, such as “disease-negative” and “disease-positive”), it ignores and/or confounds three or more biologically distinct situations (46). Because it assumes that disease prevalence is constant, the sensitivity and specificity estimates generated by the “2 x 2 table” model are not valid when disease prevalence differs. As the COVID-19 pandemic has abundantly illustrated, disease prevalence may change very rapidly, and it may grow or diminish following a quasi-exponential function (50, 52).

The “2 x 2 table” paradigm operates together with cutoff-based models, which assume that continuous data can be converted into discontinuous entities (53). **Figures 2A–D** illustrate the errors induced by the “2 x 2 table” model when it is applied with thresholds. If the highest value of each data distribution was hypothesized to be the limit that separates survivors from non-survivors, the evidence refutes such a hypothesis: a simple histogram will show a high number of misclassified observations (**Figures 2A**–**D**). While cutoff-based, binary models do not apply to infectious diseases in which three or more biological conditions may occur and disease prevalence differ across populations (and, over time, within the same population), the non-reductionist approach appears to be an alternative.

An additional problem to be prevented involves *spatial relativity* (54). This term refers to the apparent lack of relationship between space and time, which results in a large portion of the space being occupied by observations collected within a short period of time and vice versa. As observed in **Figure 9A**, data points collected over 4 days occupied a larger area of the plot than those reported over 12 days. This feature could lead to errors if predictions were based on linear models. However, when data circularity (an emergent property) and pattern recognition are considered, some clusters of data points are prognostic: observations collected at days 3 to 7 were only composed of non-survivors (**Figure 9B**).

These examples suggest that methods that capture well conserved features expressed by many vertebrate species may be more informative than reductionist analyses (15, 29, 55). In addition, the analysis of dynamic complexity may complement statistical analysis: when statistical significance was not reached, pattern recognition identified a substantial percentage of observations only associated with one outcome (**Figures 13B, D, F, H**).

### Hypothesis Generation

Two considerations emerged, which induced a question. Given that (1) the same viral variant infected both survivors and non-survivors in both populations investigated, and (2) since hospitalization day 1, patients that eventually survived differed immunologically from patients that died while hospitalized, can the virus be the only reason for the immunological difference, or could patients differ immunologically before the infection took place? The fact that survivors and non-survivors shared the pathogen does not support the hypothesis of viral led only pathogenesis. Instead, the possibility that patients who did not survive might have a different immune profile before the infection took place cannot be ruled out. Addressing such a hypothesis or any hypothesis with a method that tends to extract more information from the same data than alternatives may rapidly foster new research initiatives.

### Reproducibility

Readers can co-validate the basic concepts of the method. The validity of the construct (comparing the information generated by the classic and the alternative approach) can be demonstrated: the analysis of leukocyte counts or percentages of survivors and non-survivors (facilitated by **Supplementary Tables S2**, **S4**) can reproduce the overlapping distributions shown in **Figures 1**, **2**, **7**. Similarly, the Principal Component analysis reported in **Figure 11** can be recreated. By making three-dimensional plots of the complex indicators reported in **Supplementary Table S2**, readers can confirm that data structuring leads to more interpretable and usable information than non-structured data.

### Caveats and Conclusions

Because numerous biological differences exist within and between individuals (including disease stages when patients are hospitalized, demographic, co-morbidity- and population-related differences), several data structures (redundant analysis) should be explored, and inferences should not depend on any one data structure. To promote validity, redundancy is essential.

## Data Availability Statement

The original contributions presented in the study are included in the article/**Supplementary Material**. Further inquiries can be directed to the corresponding authors.

## Author Contributions

AR, MR, AA, FF, and JH: Methodology. AR: Software. JV, CL, GK, RK, BA, RD, and LK: Data collection and curation. CL, JV, PK, and YG: Original draft preparation. Everyone: Writing, Reviewing, and Editing. All authors contributed to the article and approved the submitted version.

## Funding

The support facilitated by the Department of Medicine of Mayo Clinic Florida (SARDOM #93960006) is appreciated. The participation of Dr. F.O. Fasina was funded by the Food and Agriculture Organization of the United Nations through USAID Grant number GHA-G-00-06-00001, Support FAO Preparedness and Response Activities to Address the Novel Global Coronavirus (COVID-19) Outbreak in Tanzania.

## Conflict of Interest

Author AA was employed by the company Stremble Ventures, LTD. Author AR is a co-inventor of the temporary guides used to recognize data patterns (European Union patent number 2959295, US patent number 10,429,389 B2).

The remaining authors declare that the research was conducted in the absence of any commercial or financial relationships that could be construed as a potential conflict of interest.

## Publisher’s Note

All claims expressed in this article are solely those of the authors and do not necessarily represent those of their affiliated organizations, or those of the publisher, the editors and the reviewers. Any product that may be evaluated in this article, or claim that may be made by its manufacturer, is not guaranteed or endorsed by the publisher.
