# Supplementary material for: Multi-Cellular Immunological Interactions Associated With COVID-19 Infections
Source: Front Immunol. 2022 Feb 24;13:794006. doi: 10.3389/fimmu.2022.794006 (PMC8913044; doi:10.3389/fimmu.2022.794006)

**Supplementary Materials**

**Multicellular immunological interactions associated with COVID-19 infections**

Jitender S. Verma^1^*, Claudia R. Libertin^2^, Yash Gupta^2^, Geetika Khanna^1^, Rohit Kumar^3^, Balvinder S. Arora^4^, Loveneesh Krishna^1^, Folorunso O. Fasina ^5^, James B. Hittner ^6^, Athos Antoniades ^7^, Marc H. V. van Regenmortel MHV^8^, Prakasha Kempaiah^2^*, Ariel L. Rivas ^9^*

^1^Central Institute of Orthopaedics, Vardhman Mahavir Medical College & Safdarjung Hospital, New Delhi, India; ^2^Infectious Diseases, Mayo Clinic, Jacksonville, FL, United States; ^3^Respiratory Medicine, Vardhman Mahavir Medical College & Safdarjung Hospital, New Delhi, India; ^4^Department of Microbiology, Vardhman Mahavir Medical College & Safdarjung Hospital, New Delhi, India; National Institute of Malaria Research, New Delhi, India; ^5^Food and Agriculture Organization of the United Nations, Dar es Salaam, United Republic of Tanzania & Department of Veterinary Tropical Diseases, University of Pretoria; ^6^College of Charleston, Charleston, SC, United States; ^7^Stremble Ventures LTD, Limassol, Cyprus; ^8^Medical University of Vienna, Austria; and Higher School of Biotechnology, University of Strasbourg; ^9^Center for Global Health-Division of Infectious Diseases, School of Medicine, University of New Mexico, Albuquerque, NM, United States

***Address correspondence:** Drs. Jitender S. Verma, Prakasha Kempaiah, and Ariel L. Rivas., e-mails: [Jitu_jitender1@rediffmail.com](mailto:Jitu_jitender1@rediffmail.com); kempaiah.prakasha[@mayo.edu](mailto:@mayo.edu); [alrivas@unm.edu](mailto:alrivas@unm.edu)

**Supplementary Tables**

| **Acronym** | **Morbidity** |
| --- | --- |
| **AE** | Acute Encephalitis |
| **AFIB** | Atrial fibrillation |
| **AG** | Acute gastroenteritis |
| **ARDS** | Acute renal distress syndrome |
| **AVR** | Aortic Valve Replacement |
| **BPH** | Benign prostatic hyperplasia |
| **bK** | Tuberculosis (Koch’s bacillus) |
| **CAD** | Coronary artery disease |
| **CHF** | Congestive heart failure |
| **CKD** | Chronic kidney failure |
| **CKD3** | Stage 3 chronic kidney disease |
| **CPAP** | Continuous positive airway pressure |
| **COPD** | Chronic obstructive pulmonary disease |
| **CRF** | Chronic renal failure |
| **CVA** | Cerebral vascular accident |
| **DCD** | Developmental Cognitive Disorder |
| **DM** | Diabetes Mellitus type 1 |
| **DM-2** | Diabetes Mellitus type 2 |
| **DVT** | Deep vein thrombosis |
| **ESRD/HD** | End-Stage Renal Disease |
| **HA** | Hemolytic Anemia |
| **HDLD** | Elevated High-Density Lipoprotein Levels |
| **HLD** | Hypersensitivity lung disease |
| **HLPD** | Hyperkeratosis lenticularis perstans |
| **HTN** | Hypertension |

**Table ST 1. Co-morbidities**

**Table ST 1 (continued)**

| **IBS** | Irritable bowel syndrome |
| --- | --- |
| **ILD** | Interstitial lung disease |
| **LRTI** | Lower respiratory tract infection |
| **Jaun** | Jaundice |
| **MA** | Metabolic acidosis |
| **OSA** | Obstructive sleep apnea |
| **PAD** | Peripheral arterial disease |
| **Panc** | Pancreatitis |
| **PE** | Pulmonary embolism |
| **Pneu** | Bi-lateral pneumonitis |
| **RA** | Rheumatoid arthritis |
| **PPM** | Permanent Pacemaker |
| **RF** | Renal failure |
| **RSA** | Right sacroanterior position |
| **SARI** | Severe acute respiratory illness |
| **S** | Sepsis |
| **SLE** | Systemic lupus erythematosus |
| **SS** | Septic shock |
| **SZ** | seizure |
| **TB** | Tuberculosis |
| **TN** | Thymic neuroendocrine tumor |
| **TNOF** | Trauma (neck of femur) |
| **UC** | Ulcerative colitis |

**Table ST 2. Population I (New Delhi study)-Demographic, clinical and immunological status and outcomes.**

| **Id^1^** | **A^2^** | **S^3^** | **Co-morbidity(ies)*** | **Outcome** | **D^4^** | **Gr^5^** | **TLC^6^** | **N %^7^** | **M %^8^** | **L %^9^** |
| --- | --- | --- | --- | --- | --- | --- | --- | --- | --- | --- |
| 5 | 84 | M | ARDS, S | Non-survivor | 1 | F | 5.40 | 52.4 | 8.1 | 39.5 |
| 6 | 75 | F | ARDS | Non-survivor | 1 | A | 8.80 | 77.9 | 4.3 | 17.8 |
| 7 | 78 | F | SARI. Thymic NET | Non-survivor | 1 | F | 2.70 | 77.0 | 2.0 | 21.0 |
| 8 | 31 | M | TB | Non-survivor | 1 | A | 6.00 | 82.0 | 4.0 | 14.0 |
| 9 | 48 | M | TB, Pneu | Non-survivor | 1 | A | 8.40 | 88.0 | 3.0 | 9.0 |
| 10 | 72 | M | HTN & DM | Non-survivor | 1 | A | 16.60 | 80.0 | 4.0 | 16.0 |
| 12 | 72 | M | SARI, HTN, DM | Non-survivor | 1 | A | 11.30 | 72.1 | 4.7 | 23.2 |
| 14 | 70 | M | SARI, DM | Non-survivor | 1 | A | 22.00 | 77.7 | 4.1 | 18.2 |
| 15 | 12 | F | AG | Non-survivor | 1 | A | 10.20 | 78.0 | 4.0 | 18.0 |
| 16 | 65 | F | HTN, DM, CKD | Non-survivor | 1 | A | 12.40 | 87.0 | 5.0 | 8.0 |
| 17 | 68 | F | CKD, CRF, SLE | Non-survivor | 1 | A | 6.90 | 64.4 | 8.2 | 27.4 |
| 18 | 48 | M | SARI | Non-survivor | 1 | F | 8.10 | 76.2 | 3.2 | 20.6 |
| 19 | 35 | M | SARI, Pneu | Non-survivor | 1 | F | 10.30 | 79.0 | 3.0 | 18.0 |
| 20 | 60 | M | CRF, DM, HTN | Non-survivor | 1 | A | 9.30 | 90.0 | 2.0 | 8.0 |
| 21 | 60 | F | Panc, SS, DM -2, ARDS | Non-survivor | 1 | F | 11.10 | 65.5 | 2.6 | 31.9 |
| 22 | 41 | M | Pneu, HTN | Non-survivor | 1 | D | 6.40 | 50.0 | 2.0 | 48.0 |
| 45 | 30 | F | Jaundice, ARDS, SS | Non-survivor | 1 | A | 25.40 | 65.2 | 8.9 | 25.9 |
| 46 | 30 | M | Pneu, MA | Non-survivor | 1 | F | 8.00 | 64.0 | 2.0 | 34.0 |
| 47 | 71 | F | Pneu, ARDS | Non-survivor | 1 | A | 19.60 | 83.3 | 7.4 | 9.3 |
| 49 | 45 | M | Pneu, ARDS | Non-survivor | 1 | B | 16.20 | 79.5 | 8.5 | 12.0 |
| 50 | 45 | M | Pneu, ARDS, DM | Non-survivor | 1 | F | 78.50 | 46.2 | 3.7 | 50.1 |
| 51 | 73 | M | Pneu, SS | Non-survivor | 1 | A | 8.60 | 86.0 | 2.0 | 12.0 |
| 1 | 56 | M | Pneu, LRTI, DM-2 | Non-survivor | 2 | F | 19.70 | 71.5 | 3.7 | 24.8 |
| 2 | 29 | M | TB, CRF | Non-survivor | 2 | A | 15.20 | 75.5 | 5.1 | 19.4 |
| 4 | 2 | M | AE, RF | Non-survivor | 2 | F | 28.50 | 63.2 | 6.6 | 30.2 |
| 11 | 31 | F | SARI | Non-survivor | 2 | F | 7.80 | 65.1 | 5.4 | 29.5 |
| 13 | 8 | F | SARI | Non-survivor | 2 | F | 11.40 | 75.2 | 2.7 | 22.1 |
| 17 | 68 | F | CKD, CRF, SLE | Non-survivor | 2 | C | 3.20 | 34.2 | 11.2 | 54.6 |
| 20 | 60 | M | CRF, DM, HTN | Non-survivor | 2 | A | 8.20 | 84.3 | 2.9 | 12.7 |
| 45 | 30 | F | Jaundice, ARDS, SS | Non-survivor | 2 | B | 33.80 | 66.0 | 14.5 | 19.5 |
| 51 | 73 | M | Pneu, SS | Non-survivor | 2 | A | 17.30 | 80.8 | 3.5 | 15.7 |
| 3 | 49 | F | ARDS, HTN | Non-survivor | 3 | A | 15.60 | 81.1 | 7.3 | 11.6 |
| 15 | 12 | F | AG | Non-survivor | 3 | F | 3.50 | 64.0 | 5.0 | 31.0 |
| 16 | 65 | F | HTN, DM, CKD | Non-survivor | 3 | A | 11.50 | 81.0 | 6.0 | 13.0 |
| 19 | 35 | M | SARI, Pneu, | Non-survivor | 3 | F | 6.90 | 74.0 | 2.0 | 24.0 |
| 20 | 60 | M | CRF, DM, HTN | Non-survivor | 3 | A | 9.50 | 83.00 | 2.0 | 15.0 |
| 21 | 60 | F | Panc, SS, DM-2, ARDS | Non-survivor | 3 | E | 24.20 | 89.2 | 6.4 | 4.4 |
| 21 | 60 | F | Panc, SS, DM-2, ARDS | Non-survivor | 3 | E | 21.20 | 89.3 | 5.0 | 5.7 |
| 45 | 30 | F | Jaundice, ARDS, SS | Non-survivor | 3 | B | 21.10 | 69.9 | 17.5 | 12.6 |
| 47 | 71 | F | Pneu, ARDS | Non-survivor | 3 | A | 15.90 | 82.9 | 5.6 | 11.5 |
| 49 | 45 | M | Pneu, ARDS | Non-survivor | 3 | A | 5.10 | 73.7 | 5.3 | 21.0 |
| 50 | 45 | M | Pneu, ARDS, DM | Non-survivor | 3 | C | 57.80 | 27.8 | 5.1 | 67.1 |
| 51 | 73 | M | Pneu, SS | Non-survivor | 3 | A | 21.80 | 90.0 | 2.0 | 8.0 |
| 13 | 8 | F | SARI | Non-survivor | 4 | A | 10.90 | 80.3 | 7.2 | 12.5 |

**Table ST 2 (continued).**

| **Id^1^** | **A^2^** | **S^3^** | **Co-morbidity(ies)*** | **Outcome** | **D^4^** | **Gr^5^** | **TLC^6^** | **N %^7^** | **M %^8^** | **L %^9^** |
| --- | --- | --- | --- | --- | --- | --- | --- | --- | --- | --- |
| 16 | 65 | F | HTN, DM, CKD | Non-survivor | 4 | F | 23.00 | 76.8 | 3.5 | 19.7 |
| 20 | 60 | M | CRF, DM, HTN | Non-survivor | 4 | A | 5.10 | 75.0 | 6.0 | 19.0 |
| 45 | 30 | F | Jaundice, ARDS, SS | Non-survivor | 4 | B | 23.30 | 65.8 | 22.8 | 11.4 |
| 48 | 38 | F | trauma# (R)NOF | Non-survivor | 4 | A | 15.50 | 79.4 | 6.1 | 14.5 |
| 48 | 38 | F | trauma# (R)NOF | Non-survivor | 4 | A | 21.40 | 81.6 | 7.2 | 11.2 |
| 50 | 45 | M | Pneu, ARDS, DM | Non-survivor | 4 | C | 63.80 | 33.3 | 5.3 | 61.4 |
| 4 | 2 | M | AE, RF | Non-survivor | 5 | C | 9.10 | 30.48 | 4.61 | 64.9 |
| 11 | 31 | F | SARI | Non-survivor | 5 | A | 7.20 | 75.0 | 6.2 | 18.8 |
| 16 | 65 | F | HTN, DM, CKD | Non-survivor | 5 | A | 12.40 | 73.6 | 8.2 | 18.2 |
| 19 | 35 | M | SARI, Pneu | Non-survivor | 5 | F | 3.30 | 83.0 | 2.0 | 15.0 |
| 20 | 60 | M | CRF, DM, HTN | Non-survivor | 5 | A | 5.00 | 83.0 | 3.0 | 14.0 |
| 21 | 60 | F | Panc, SS, DM-2, ARDS | Non-survivor | 5 | A | 18.50 | 78.9 | 6.0 | 15.1 |
| 45 | 30 | F | Jaundice, ARDS, SS | Non-survivor | 5 | B | 21.10 | 66.6 | 21.0 | 12.4 |
| 47 | 71 | F | Pneu, ARDS | Non-survivor | 5 | F | 14.20 | 73.9 | 2.2 | 23.9 |
| 48 | 38 | F | trauma# (R)NOF | Non-survivor | 5 | B | 7.80 | 78.85 | 9.81 | 11.3 |
| 51 | 73 | M | Pneu, SS | Non-survivor | 5 | E | 36.40 | 90.5 | 4.1 | 5.4 |
| 13 | 8 | F | SARI | Non-survivor | 6 | A | 13.40 | 77.6 | 5.7 | 16.7 |
| 16 | 65 | F | HTN, DM, CKD | Non-survivor | 6 | A | 8.90 | 76.8 | 6.6 | 16.6 |
| 45 | 30 | F | Jaundice, ARDS, SS | Non-survivor | 6 | B | 16.80 | 54.5 | 24.8 | 20.7 |
| 50 | 45 | M | Pneu, ARDS, DM | Non-survivor | 6 | C | 66.90 | 35.0 | 4.3 | 60.7 |
| 2 | 29 | M | TB, CRF | Non-survivor | 7 | A | 5.00 | 65.3 | 8.1 | 26.6 |
| 16 | 65 | F | HTN, DM, CKD | Non-survivor | 7 | E | 14.20 | 92.6 | 2.2 | 5.2 |
| 47 | 71 | F | Pneu, ARDS | Non-survivor | 7 | A | 13.90 | 83.1 | 3.5 | 13.4 |
| 48 | 38 | F | trauma# (R)NOF | Non-survivor | 7 | B | 6.10 | 75.8 | 13.3 | 10.9 |
| 50 | 45 | M | Pneu, ARDS, DM | Non-survivor | 7 | C | 57.00 | 33.2 | 4.6 | 62.2 |
| 47 | 71 | F | Pneu, ARDS | Non-survivor | 8 | A | 13.20 | 77.1 | 5.5 | 17.4 |
| 4 | 2 | M | AE, RF | Non-survivor | 9 | F | 9.10 | 44.6 | 4.0 | 51.4 |
| 2 | 29 | M | TB, CRF | Non-survivor | 12 | F | 7.50 | 57.5 | 2.9 | 39.6 |
| 4 | 2 | M | AE, RF | Non-survivor | 12 | F | 9.10 | 58.7 | 7.2 | 34.1 |
| 23 | 25 | F | PNEU, HTN | Survivor | 1 | F | 7.90 | 67.5 | 4.3 | 28.2 |
| 24 | 24 | F | PNEU, HTN | Survivor | 1 | F | 11.00 | 69.4 | 3.2 | 27.4 |
| 25 | 45 | F | Travel history | Survivor | 1 | F | 5.10 | 66.3 | 4.5 | 29.2 |
| 26 | 16 | M | Contact history | Survivor | 1 | F | 3.10 | 48.6 | 3.4 | 48.0 |
| 27 | 35 | M | Contact history | Survivor | 1 | D | 4.40 | 45.0 | 2.0 | 53.0 |
| 28 | 36 | F | HTN, DM | Survivor | 1 | F | 8.50 | 62.0 | 3.0 | 35.0 |

**Table ST 2 (continued).**

| **Id**^1^ | **A**^2^ | **S**^3^ | **Co-morbidity(ies)*** | **Outcome** | **D**^4^ | **Gr**^5^ | **TLC**^6^ | **N %**^7^ | **M %**^8^ | **L %**^9^ |
| --- | --- | --- | --- | --- | --- | --- | --- | --- | --- | --- |
| 29 | 44 | M | HTN | Survivor | 1 | F | 6.50 | 58.0 | 4.0 | 38.0 |
| 30 | 42 | M | HTN | Survivor | 1 | F | 4.70 | 58.2 | 3.6 | 38.2 |
| 31 | 38 | M | Travel history | Survivor | 1 | F | 4.30 | 49.4 | 2.9 | 47.7 |
| 32 | 36 | M | Travel history | Survivor | 1 | F | 4.90 | 62.5 | 3.9 | 33.6 |
| 33 | 45 | M | Travel history | Survivor | 1 | F | 5.80 | 59.0 | 5.4 | 35.6 |
| 34 | 22 | F | Travel history | Survivor | 1 | F | 9.30 | 74.3 | 3.6 | 22.1 |
| 35 | 25 | M | Travel history | Survivor | 1 | D | 5.60 | 60.4 | 1.6 | 38.0 |
| 36 | 38 | M | Travel history, DM-2 | Survivor | 1 | F | 4.70 | 52.0 | 5.6 | 42.4 |
| 37 | 28 | M | Travel history | Survivor | 1 | F | 6.00 | 54.3 | 7.5 | 38.2 |
| 38 | 32 | M | Travel history | Survivor | 1 | F | 5.50 | 66.0 | 4.0 | 30.0 |
| 39 | 25 | F | Travel history | Survivor | 1 | D | 4.80 | 57.6 | 1.0 | 41.4 |
| 40 | 25 | M | Travel history | Survivor | 1 | F | 11.40 | 59.0 | 7.4 | 33.6 |
| 41 | 41 | M | Travel history | Survivor | 1 | F | 6.70 | 56.4 | 7.1 | 36.5 |
| 42 | 35 | M | Travel history | Survivor | 1 | F | 5.60 | 50.4 | 9.2 | 40.4 |
| 43 | 24 | F | Contact history | Survivor | 1 | F | 5.70 | 52.8 | 5.2 | 42.0 |
| 44 | 65 | M | PNEU | Survivor | 1 | A | 8.30 | 85.0 | 3.6 | 11.4 |
| 38 | 32 | M | Travel history | Survivor | 3 | F | 3.50 | 61.4 | 2.1 | 36.5 |
| 40 | 25 | M | Travel history | Survivor | 4 | A | 9.90 | 59.5 | 10.2 | 30.2 |
| 25 | 45 | F | Travel history | Survivor | 5 | F | 4.60 | 69.2 | 2.8 | 28.0 |

^1^: patient identifier; ^2^: age; ^3^: gender; ^4^: day[s] post-admission; ^5^: patient group (A-E); ^6^: total leukocyte count/microliter; ^7^: neutrophil; ^8^: lymphocyte; ^9^: monocyte. * See description of each co-morbidity in Table 1.

**Table ST 3. Population I - Imunological profiles of patient subsets**

| As shown by spatial patterns | these patient groups were distinguished | which showed values predominantly non-overlapping of the |
| --- | --- | --- |
| *AAT, BBE* | A and B | M % |
| *BAG, AAE, AW* | A and C | L % |
| *AX/AZ, P, AAW* | A and D | ([L/M/M/L] / [P/MC]) ratio |
| *BAX, AW, T* | A and E | N % |
| *T, P, AX/AZ* | A and F | ([L/M/M/L] / [P/MC]) ratio |
|  |  |  |
| *AAS/AAR, S, BAG/AAE* | B and C | L % |
| *AAU/AAA, BAG/AAT* | B and D | L %, M % |
| *AW/AX, BAU/BBE* | B and E | M % |
| *BBE/AAW, BAN/BBE* | B and F | ([L/M/M/L] / [P/MC]) ratio |
|  |  |  |
| *AZ, T/S/P* | C and D | L %, N %, M % |
| *AW/AX/AZ, AAU/AAA/AAD* | C and E | L %, N % |
| *AAE/AAT, T/S/P* | C and F | L %, N % |
|  |  |  |
| *AW/AAB* | D and E | L %, N % |
| *P, AAW* | D and F | ([L/M/M/L] / [P/MC]) ratio |
|  |  |  |
| *BAN/BBE/BAX* | E and F | L %, N % |

- Letters shown in italics do not have any specific biological meaning. These identifiers are dimensionless numbers that may express relationships (usually, functions not previously been investigated and/or reported in the literature), which are performed by two or more cell types.
- Groups A-E: groups of patients that, in three-dimensional (3D) space, exhibit at least one distinct data pattern (e.g., a perpendicular data inflection). Groups of patients are not determined by any one value of any one data combination (a dimensionless number) but by the pattern(s) shown in 3D space. Because it is the point in space where the three variables measured in a 3D plot converge what determines pattern recognition, no pattern can be predicted from the analysis of any one dimensionless number. This process is expected to reflect the numerous and changing functions performed, *in vivo*, by multicellularity. Because this analytical process may induce artifacts, a biologically interpretable validation is conducted considering two or more leukocyte data distributions of times. Patient data partitioning is regarded as plausible when two or more biologically meaningful classes (e.g., different outcomes or different co-morbidities) display (predominanrly or totally) non-overlapping distributions of leukocyte percentages or ratios. For example, patient groups A and C displayed non-overlapping lymphocyte percentages.
- L, N, or M: lymphocytes, neutrophils, or monocytes, respectively
- P: phagocytes (neutrophils and monocytes)
- M C: mononuclear cells (lymphocytes and monocytes)

**Table ST 4. Population II (Jacksonville study)-Demographic, clinical and immunological status and outcomes.**

| **Id**^1^ | **A**^2^ | **S**^3^ | **Co-morbidity(ies)*** | **Outcome** | **D**^4^ | **TLC**^6^ | **N %**^7^ | **M %**^8^ | **L %**^9^ |
| --- | --- | --- | --- | --- | --- | --- | --- | --- | --- |
| B36 | 78 | M | CKD3, DM, COPD | Non-survivor | 1 | 4.90 | 91.3 | 2.4 | 6.1 |
| B3 | 73 | M | DM, CAD, HLPD, HTN | Non-survivor | 1 | 13.80 | 93.3 | 5.0 | 1.6 |
| B4 | 77 | M | DM, HTN | Non-survivor | 1 | 7.60 | 81.1 | 2.2 | 16.6 |
| B7 | 56 | M | HTN | Non-survivor | 1 | 9.20 | 89.8 | 5.3 | 4.8 |
| B1 | 91 | F | CAD, ILD, CKD3 | Non-survivor | 1 | 4.90 | 83.9 | 3.9 | 12.0 |
| B66 | 68 | F | RA, interstitial lung | Non-survivor | 1 | 2.90 | 85.8 | 6.9 | 7.2 |
| B2 | 64 | F | PE | Non-survivor | 1 | 11.10 | 84.7 | 6.4 | 8.8 |
| B14 | 44 | M | None | Non-survivor | 1 | 6.50 | 77.9 | 4.8 | 17.2 |
| B6 | 71 | F | DCD, panic disorder | Non-survivor | 1 | 13.70 | 77.6 | 7.7 | 14.5 |
| B75 | 83 | M | AFIB, HTN, HPLD, PE | Non-survivor | 1 | 3.10 | 72.3 | 8.0 | 19.5 |
| B65 | 67 | F | UC, HTN, HPLD, CAD | Non-survivor | 1 | 6.20 | 70.6 | 7.5 | 21.7 |
| B72 | 88 | M | DM, HTN, Afib, PPM | Non-survivor | 1 | 6.50 | 75.7 | 12.8 | 11.4 |
| B71 | 87 | F | COPD, chronic pain | Non-survivor | 1 | 6.50 | 68.9 | 10.1 | 20.9 |
| B3 | 73 | M | DM, CAD, HLPD, HTN | Non-survivor | 2 | 12.10 | 93.4 | 4.8 | 1.7 |
| B4 | 77 | M | DM, HTN | Non-survivor | 2 | 11.70 | 91.4 | 4.0 | 4.4 |
| B7 | 56 | M | HTN | Non-survivor | 2 | 9.30 | 90.3 | 3.9 | 5.7 |
| B6 | 71 | F | DCD, panic disorder | Non-survivor | 2 | 13.80 | 83.6 | 2.5 | 13.8 |
| B36 | 78 | M | CKD3, DM, COPD | Non-survivor | 2 | 4.60 | 88.1 | 4.4 | 7.4 |
| B66 | 68 | F | RA, interstitial lung | Non-survivor | 2 | 3.90 | 85.7 | 8.2 | 5.9 |
| B2 | 64 | F | PE | Non-survivor | 2 | 7.40 | 77.6 | 5.1 | 17.1 |
| B1 | 91 | F | CAD, ILD, CKD4 | Non-survivor | 2 | 5.60 | 80.1 | 6.2 | 13.6 |
| B72 | 88 | M | DM, HTN, Afib, PPM | Non-survivor | 2 | 3.70 | 76.6 | 9.3 | 14.0 |
| B65 | 67 | F | UC, HTN, HPLD, CAD | Non-survivor | 2 | 3.80 | 60.5 | 7.4 | 32.0 |
| B14 | 44 | M | None | Non-survivor | 2 | 3.40 | 56.9 | 6.7 | 36.3 |
| B3 | 73 | M | DM, CAD, HLPD, HTN | Non-survivor | 3 | 12.80 | 93.6 | 4.0 | 2.3 |
| B36 | 78 | M | CKD 3, DM, COPD | Non-survivor | 3 | 4.00 | 90.2 | 3.5 | 6.2 |
| B66 | 68 | F | RA, interstitial lung | Non-survivor | 3 | 4.20 | 88.2 | 5.7 | 6.0 |
| B6 | 71 | F | DCD, panic disorder | Non-survivor | 3 | 15.40 | 82.5 | 4.6 | 12.8 |
| B2 | 64 | F | PE | Non-survivor | 3 | 8.40 | 82.0 | 4.7 | 13.1 |
| B1 | 91 | F | CAD, ILD, CKD5 | Non-survivor | 3 | 7.60 | 80.5 | 7.1 | 12.2 |
| B65 | 67 | F | DMW, HTN, HLPD | Non-survivor | 3 | 4.20 | 69.3 | 6.5 | 24.0 |
| B14 | 44 | M | None | Non-survivor | 3 | 14.60 | 71.8 | 9.0 | 19.2 |
| B72 | 88 | M | DM, HTN, Afib, PPM | Non-survivor | 3 | 3.70 | 69.7 | 9.0 | 21.1 |
| B7 | 56 | M | HTN | Non-survivor | 3 | 9.00 | 70.0 | 10.1 | 19.7 |
| B3 | 73 | M | DM, CAD, HLPD, HTN | Non-survivor | 4 | 13.00 | 94.8 | 3.1 | 2.0 |
| B2 | 64 | F | PE | Non-survivor | 4 | 14.00 | 92.6 | 2.8 | 4.4 |
| B2 | 64 | F | PE | Non-survivor | 5 | 13.80 | 89.4 | 2.6 | 7.8 |
| B70 | 62 | M | ESRD/HD, HTN, DM | Survivor | 1 | 7.90 | 93.4 | 2.2 | 4.3 |
| B15 | 75 | M | HTN, lipids | Survivor | 1 | 10.50 | 94.6 | 3.2 | 2.1 |
| B10 | 74 | F | DM, anxiety, ILD, CHF | Survivor | 1 | 14.40 | 88.5 | 1.4 | 10.0 |
| B20 | 50 | M | Latent TB | Survivor | 1 | 6.90 | 90.2 | 1.9 | 7.8 |

**Table ST 4 (continued).**

| **Id**^1^ | **A**^2^ | **S**^3^ | **Co-morbidity(ies)*** | **Outcome** | **D**^4^ | **TLC**^6^ | **N %**^7^ | **M %**^8^ | **L %**^9^ |
| --- | --- | --- | --- | --- | --- | --- | --- | --- | --- |
| B67 | 62 | M | IBS | Survivor | 1 | 6.00 | 90.5 | 2.0 | 7.4 |
| B16 | 53 | M | Obesity | Survivor | 1 | 9.40 | 92.8 | 2.8 | 4.3 |
| B5 | 60 | M | OSA, CPAP | Survivor | 1 | 8.60 | 89.1 | 1.9 | 8.8 |
| B82 | 61 | F | Asthma, DM | Survivor | 1 | 8.60 | 89.1 | 1.9 | 8.8 |
| B85 | 85 | M | ILD, DM, HTN, HLD,CVA | Survivor | 1 | 6.20 | 89.1 | 1.9 | 8.8 |
| A9 | 56 | M | None | Survivor | 1 | 13.20 | 93.3 | 4.1 | 2.5 |
| B26 | 60 | F | Hypothyroidism | Survivor | 1 | 8.10 | 87.9 | 4.2 | 7.8 |
| B28 | 50 | M | Kidney stone | Survivor | 1 | 10.10 | 84.6 | 3.1 | 12.2 |
| A13 | 75 | M | Gerd, HA, hypothyroidism | Survivor | 1 | 9.00 | 88.0 | 4.8 | 7.0 |
| B19 | 61 | M | SZ, DVT, CAD, COPD | Survivor | 1 | 5.70 | 86.2 | 4.6 | 9.1 |
| A10 | 71 | F | None | Survivor | 1 | 7.20 | 83.9 | 4.3 | 11.7 |
| B37 | 54 | F | DM, asthma | Survivor | 1 | 5.30 | 86.2 | 5.5 | 8.2 |
| B64 | 69 | M | UC, Inc lft, | Survivor | 1 | 12.20 | 86.8 | 6.8 | 6.3 |
| B34 | 42 | F | Asthma, COPD, HTN | Survivor | 1 | 2.30 | 79.5 | 3.9 | 16.5 |
| B33 | 51 | M | Asthma, HTN, OSA | Survivor | 1 | 10.70 | 86.2 | 7.1 | 6.5 |
| B35 | 69 | M | Pit adenoma, CAD | Survivor | 1 | 8.10 | 84.5 | 6.2 | 9.1 |
| A17 | 88 | F | COPD, dementia | Survivor | 1 | 8.90 | 85.6 | 8.1 | 6.2 |
| B47 | 62 | M | NONE | Survivor | 1 | 4.00 | 80.8 | 5.8 | 13.3 |
| B11 | 66 | F | HTN, HLD | Survivor | 1 | 5.10 | 79.7 | 5.4 | 14.8 |
| B21 | 51 | M | None | Survivor | 1 | 9.50 | 84.8 | 8.3 | 6.8 |
| B17 | 38 | M | Obesity, ex smoker | Survivor | 1 | 5.60 | 77.7 | 4.9 | 17.3 |
| B60 | 59 | M | None | Survivor | 1 | 7.30 | 82.9 | 7.7 | 9.3 |
| A7 | 63 | M | HTN, skin melanoma | Survivor | 1 | 8.10 | 81.4 | 7.0 | 11.5 |
| B52 | 48 | M | None | Survivor | 1 | 4.00 | 76.9 | 5.4 | 17.6 |
| B9 | 56 | F | HA, HLDP | Survivor | 1 | 8.30 | 81.8 | 8.1 | 9.9 |
| B41 | 59 | M | ESRD/HD, CAD, CVA | Survivor | 1 | 10.60 | 80.6 | 7.5 | 11.8 |
| B55 | 50 | M | HTN, CAD, OSA, HDLD | Survivor | 1 | 8.70 | 78.0 | 6.4 | 15.5 |
| B30 | 39 | F | Bi-polar disorder | Survivor | 1 | 6.80 | 76.8 | 5.9 | 17.1 |
| B69 | 51 | M | None | Survivor | 1 | 6.20 | 81.9 | 8.7 | 9.2 |
| B50 | 92 | M | DVT, COPD, CKD | Survivor | 1 | 6.10 | 81.5 | 8.7 | 9.7 |
| B25 | 33 | M | None | Survivor | 1 | 3.70 | 75.2 | 5.6 | 19.1 |
| A23 | 48 | M | DM | Survivor | 1 | 4.40 | 80.5 | 8.2 | 11.2 |
| B53 | 45 | M | None | Survivor | 1 | 5.30 | 72.8 | 4.9 | 22.1 |
| B38 | 65 | F | None | Survivor | 1 | 5.30 | 74.1 | 5.5 | 20.3 |
| B18 | 50 | F | None | Survivor | 1 | 4.10 | 75.8 | 6.4 | 17.7 |
| A21 | 64 | M | HRN, depression | Survivor | 1 | 6.00 | 76.7 | 7.2 | 16.0 |
| B29 | 79 | M | HYN, DM, HLPD | Survivor | 1 | 8.90 | 81.8 | 10.7 | 7.3 |
| B31 | 39 | F | DM | Survivor | 1 | 7.10 | 75.3 | 6.8 | 17.8 |
| B12 | * | M | Gout, HTN | Survivor | 1 | 5.40 | 73.2 | 6.1 | 20.6 |
| B46 | 56 | M | HPLD, Lyme hx, migraine | Survivor | 1 | 5.70 | 84.4 | 14.4 | 1.0 |

**Table ST 4 (continued).**

| **Id**^1^ | **A**^2^ | **S**^3^ | **Co-morbidity(ies)*** | **Outcome** | **D**^4^ | **TLC**^6^ | **N %**^7^ | **M %**^8^ | **L %**^9^ |
| --- | --- | --- | --- | --- | --- | --- | --- | --- | --- |
| B58 | 58 | M | Hypothyroidism | Survivor | 1 | 12.00 | 79.8 | 10.2 | 9.8 |
| B59 | 57 | M | None | Survivor | 1 | 4.60 | 74.6 | 7.2 | 18.0 |
| B78 | 68 | M | None | Survivor | 1 | 4.30 | 78.3 | 9.5 | 12.0 |
| B49 | 70 | M | OSA,BPH, Inc lft | Survivor | 1 | 6.80 | 77.3 | 9.4 | 13.1 |
| B51 | 62 | M | Afib, DM2, HTN | Survivor | 1 | 7.40 | 77.8 | 10.1 | 12.0 |
| A18 | 47 | M | Asthma, OSA | Survivor | 1 | 6.70 | 78.0 | 10.3 | 11.6 |
| B38 | 70 | F | CAD,PAD, COPD | Survivor | 1 | 9.50 | 73.3 | 7.5 | 19.0 |
| B61 | 57 | M | CKD3, HTN, DM2 | Survivor | 1 | 6.10 | 76.7 | 9.5 | 13.7 |
| A11 | 77 | M | OSA, CAD, HTN | Survivor | 1 | 7.90 | 79.5 | 11.7 | 8.7 |
| A24 | 53 | F | Asthma, HTN, HLDP | Survivor | 1 | 3.60 | 70.3 | 7.0 | 22.6 |
| B62 | 61 | F | None | Survivor | 1 | 9.00 | 69.7 | 7.2 | 23.0 |
| B79 | 65 | F | HTN, DM, HPLD | Survivor | 1 | 4.80 | 71.4 | 8.6 | 19.9 |
| B81 | 82 | F | H/O breast ca - ned, | Survivor | 1 | 7.10 | 66.9 | 6.6 | 26.4 |
| B83 | 56 | F | Glaucoma, soft tiss inf | Survivor | 1 | 6.50 | 66.5 | 7.5 | 25.8 |
| B40 | 64 | M | None | Survivor | 1 | 3.60 | 68.3 | 8.4 | 23.2 |
| B57 | 64 | F | OSA, anxiety | Survivor | 1 | 3.60 | 64.7 | 7.2 | 27.9 |
| A8 | 57 | M | HTN, h/o SAH | Survivor | 1 | 6.50 | 71.7 | 12.1 | 16.1 |
| A16 | 33 | M | None | Survivor | 1 | 7.70 | 70.4 | 12.7 | 16.8 |
| B54 | 38 | M | Marfans syndrome, SZ | Survivor | 1 | 4.00 | 64.6 | 9.1 | 26.2 |
| B24 | 34 | F | Obesity | Survivor | 1 | 8.10 | 65.5 | 9.7 | 24.6 |
| B43 | 42 | M | COPD | Survivor | 1 | 5.50 | 68.3 | 11.9 | 19.7 |
| B32 | 73 | F | Hypothyroidism | Survivor | 1 | 3.60 | 59.0 | 6.9 | 34.0 |
| A20 | 65 | F | CAD, DM, Afib, CHF | Survivor | 1 | 7.60 | 69.5 | 13.1 | 17.3 |
| B27 | 42 | F | HTN | Survivor | 1 | 5.50 | 66.0 | 11.1 | 22.8 |
| B39 | 70 | M | Afib, asthma, DM | Survivor | 1 | 7.80 | 70.5 | 15.5 | 13.8 |
| B8 | 98 | F | None | Survivor | 1 | 8.20 | 66.3 | 12.5 | 21.1 |
| B48 | 56 | M | None | Survivor | 1 | 6.00 | 66.3 | 14.1 | 19.5 |
| B42 | 56 | M | None | Survivor | 1 | 6.00 | 66.1 | 14.2 | 19.6 |
| B44 | 50 | F | None | Survivor | 1 | 4.90 | 64.0 | 12.5 | 23.4 |
| A15 | 49 | M | None | Survivor | 1 | 4.50 | 57.2 | 8.7 | 33.9 |
| A14 | 92 | F | Afib, PPM, HTN,HLD | Survivor | 1 | 5.40 | 65.9 | 15.4 | 18.6 |
| A22 | 76 | F | Cirrhosis | Survivor | 1 | 4.40 | 63.4 | 15.2 | 21.3 |
| B56 | 50 | F | Bi-polar disorder | Survivor | 1 | 5.90 | 55.0 | 10.2 | 34.7 |
| B73 | 91 | M | DM, HTN, HPLD | Survivor | 1 | 4.10 | 54.4 | 10.1 | 35.4 |
| B77 | 70 | M | Anemia, UC | Survivor | 1 | 6.30 | 59.9 | 14.0 | 26.0 |
| B22 | 51 | M | DM, HTN, HPLD | Survivor | 1 | 5.40 | 51.2 | 9.4 | 39.2 |
| B74 | 56 | M | None | Survivor | 1 | 4.40 | 60.9 | 20.4 | 18.6 |
| B80 | 57 | F | HTN, DM, HPLD | Survivor | 1 | 6.20 | 54.0 | 13.7 | 32.1 |
| B63 | 46 | M | HTN, CAD, HPLD | Survivor | 1 | 9.10 | 46.0 | 11.0 | 42.8 |
| B23 | 43 | F | Depression | Survivor | 1 | 1.50 | 44.6 | 12.6 | 42.6 |
| B76 | 47 | M | None | Survivor | 1 | 4.30 | 43.4 | 20.0 | 36.4 |

**Table ST 4 (continued).**

| **Id**^1^ | **A**^2^ | **S**^3^ | **Co-morbidity(ies)*** | **Outcome** | **D**^4^ | **TLC**^6^ | **N %**^7^ | **M %**^8^ | **L %**^9^ |
| --- | --- | --- | --- | --- | --- | --- | --- | --- | --- |
| A19 | 79 | F | AFIB, HTN | Survivor | 1 | 4.00 | 42.5 | 20.5 | 36.8 |
| B70 | 62 | M | ESRD/HD, HTN, DM | Survivor | 2 | 7.20 | 96.6 | 1.1 | 2.2 |
| A9 | 56 | M | None | Survivor | 2 | 10.90 | 93.5 | 2.3 | 4.1 |
| B10 | 74 | F | DM, anxiety, ILD | Survivor | 2 | 12.80 | 91.3 | 2.0 | 6.6 |
| A17 | 88 | F | COPD, dementia | Survivor | 2 | 7.70 | 92.2 | 3.0 | 4.7 |
| B16 | 53 | M | Obesity | Survivor | 2 | 5.90 | 89.9 | 2.7 | 7.3 |
| B67 | 62 | M | IBS | Survivor | 2 | 4.00 | 89.5 | 2.7 | 7.7 |
| B15 | 75 | M | HTN,lipids | Survivor | 2 | 10.50 | 89.8 | 3.1 | 7.0 |
| B85 | 85 | M | ILD, DM, HTN, HLD | Survivor | 2 | 5.50 | 88.5 | 3.5 | 7.9 |
| B5 | 60 | M | OSA, CPAP | Survivor | 2 | 7.70 | 88.4 | 3.5 | 7.9 |
| B82 | 61 | F | Asthma, DM | Survivor | 2 | 7.70 | 88.4 | 3.5 | 7.9 |
| B51 | 62 | M | Afib, DM2, HTN, CAD | Survivor | 2 | 7.60 | 89.9 | 4.8 | 5.2 |
| B69 | 51 | M | None | Survivor | 2 | 5.50 | 83.4 | 2.8 | 13.6 |
| B20 | 50 | F | Latent TB | Survivor | 2 | 7.30 | 85.9 | 3.6 | 10.4 |
| A23 | 48 | M | DM | Survivor | 2 | 8.90 | 82.8 | 2.8 | 14.2 |
| B13 | 70 | F | DM, IBS, RA | Survivor | 2 | 5.40 | 85.6 | 3.9 | 10.4 |
| B55 | 50 | M | HTN, CAD, OSA, HDLD | Survivor | 2 | 8.80 | 80.7 | 3.0 | 16.1 |
| A13 | 75 | M | Gerd, HA | Survivor | 2 | 5.50 | 87.6 | 5.8 | 6.4 |
| A10 | 71 | F | None | Survivor | 2 | 6.00 | 83.4 | 4.0 | 12.5 |
| B29 | 79 | M | HYN, DM, HLPDh | Survivor | 2 | 5.50 | 87.0 | 5.7 | 7.2 |
| B19 | 61 | M | SZ, DVT, CAD, COPD | Survivor | 2 | 5.10 | 86.0 | 5.8 | 8.0 |
| B64 | 69 | M | UC, HTN, HPLD, CAD | Survivor | 2 | 9.40 | 84.9 | 8.8 | 6.2 |
| A7 | 63 | M | HTN, skin melanoma | Survivor | 2 | 8.30 | 83.6 | 8.0 | 8.2 |
| B35 | 69 | M | Pit adenoma, CAD | Survivor | 2 | 5.90 | 81.7 | 6.9 | 11.2 |
| B12 | * | M | Gout, HTN | Survivor | 2 | 6.20 | 74.7 | 4.2 | 21.0 |
| B61 | 57 | M | CKD3, HTN, DM2 | Survivor | 2 | 8.90 | 82.5 | 7.5 | 9.9 |
| B21 | 51 | M | None | Survivor | 2 | 6.20 | 80.5 | 7.4 | 11.9 |
| B34 | 42 | F | Asthma, COPD, HTN | Survivor | 2 | 4.10 | 73.9 | 5.1 | 20.9 |
| B58 | 58 | M | Hypothyroidism | Survivor | 2 | 7.20 | 79.0 | 7.4 | 13.4 |
| B25 | 33 | M | None | Survivor | 2 | 3.50 | 76.9 | 6.6 | 16.3 |
| B83 | 56 | F | Glaucoma, soft tiss inf | Survivor | 2 | 8.90 | 78.8 | 7.9 | 13.1 |
| A15 | 49 | M | None | Survivor | 2 | 9.10 | 79.6 | 8.5 | 11.8 |
| B31 | 39 | F | DM | Survivor | 2 | 6.50 | 74.6 | 6.4 | 18.8 |
| B37 | 54 | F | DM, asthma | Survivor | 2 | 3.20 | 75.7 | 7.1 | 17.1 |
| B17 | 38 | M | Obesity, ex smoker | Survivor | 2 | 5.70 | 72.3 | 6.1 | 21.4 |
| B60 | 59 | M | None | Survivor | 2 | 8.60 | 79.4 | 10.1 | 10.4 |
| B46 | 56 | M | HPLD, Lyme hx | Survivor | 2 | 6.70 | 83.8 | 14.6 | 1.5 |
| B26 | 60 | F | Hypothyroidism | Survivor | 2 | 5.10 | 70.8 | 5.9 | 23.2 |
| B50 | 92 | M | DVT, COPD, CKD | Survivor | 2 | 6.10 | 77.2 | 9.1 | 13.5 |
| B78 | 68 | M | None | Survivor | 2 | 5.00 | 78.0 | 9.9 | 11.9 |

**Table ST 4 (continued).**

| **Id**^1^ | **A**^2^ | **S**^3^ | **Co-morbidity(ies)*** | **Outcome** | **D**^4^ | **TLC**^6^ | **N %**^7^ | **M %**^8^ | **L %**^9^ |
| --- | --- | --- | --- | --- | --- | --- | --- | --- | --- |
| B49 | 70 | M | OSA, BPH, Inc lft | Survivor | 2 | 7.40 | 74.2 | 9.4 | 16.3 |
| A11 | 77 | M | OSA, CAD, HTN | Survivor | 2 | 9.30 | 76.4 | 11.7 | 11.7 |
| B47 | 62 | M | None | Survivor | 2 | 5.10 | 75.4 | 11.3 | 13.2 |
| A21 | 64 | M | HRN, depression | Survivor | 2 | 6.20 | 71.0 | 8.6 | 20.3 |
| B38 | 65 | F | None | Survivor | 2 | 6.00 | 67.4 | 7.1 | 25.3 |
| B32 | 73 | F | Hypothyroidism | Survivor | 2 | 7.20 | 67.3 | 7.5 | 25.1 |
| A18 | 47 | M | Asthma, OSA | Survivor | 2 | 3.90 | 71.6 | 10.4 | 17.9 |
| B38 | 70 | F | CAD, PAD, COPD | Survivor | 2 | 5.50 | 65.2 | 7.3 | 27.3 |
| B52 | 48 | M | None | Survivor | 2 | 4.30 | 64.9 | 7.3 | 27.6 |
| B33 | 51 | M | Asthma, HTN, OSA | Survivor | 2 | 4.40 | 71.3 | 10.9 | 17.6 |
| B18 | 50 | F | None | Survivor | 2 | 4.40 | 66.1 | 8.1 | 25.7 |
| B41 | 59 | M | ESRD/HD, CAD | Survivor | 2 | 6.90 | 71.1 | 11.3 | 17.5 |
| A24 | 53 | F | Asthma, HTN, HLDP | Survivor | 2 | 6.30 | 60.5 | 6.7 | 32.6 |
| B27 | 42 | F | HTN | Survivor | 2 | 7.30 | 67.5 | 10.6 | 21.8 |
| B57 | 64 | F | OSA, anxiety | Survivor | 2 | 5.10 | 61.5 | 7.5 | 30.8 |
| B42 | 56 | M | None | Survivor | 2 | 4.40 | 72.2 | 14.9 | 12.8 |
| B48 | 56 | M | None | Survivor | 2 | 5.80 | 72.2 | 14.9 | 12.8 |
| B62 | 61 | F | None | Survivor | 2 | 4.60 | 62.0 | 8.8 | 29.1 |
| A8 | 57 | M | HTN, h/o SAH | Survivor | 2 | 4.90 | 57.4 | 6.9 | 35.5 |
| B53 | 45 | M | None | Survivor | 2 | 4.00 | 61.2 | 8.9 | 29.8 |
| B30 | 39 | F | Bi-polar disorder | Survivor | 2 | 7.10 | 63.9 | 10.6 | 25.4 |
| B54 | 38 | M | AVR, CVA, SZ | Survivor | 2 | 5.70 | 63.0 | 10.1 | 26.8 |
| B43 | 42 | M | COPD | Survivor | 2 | 5.50 | 65.4 | 11.9 | 22.5 |
| A14 | 92 | F | Afib, PPM, HTN,HLD | Survivor | 2 | 4.70 | 69.5 | 15.6 | 14.8 |
| B39 | 70 | M | Afib, asthma, DM | Survivor | 2 | 4.30 | 67.4 | 14.4 | 18.1 |
| B28 | 50 | M | Kidney stone | Survivor | 2 | 3.90 | 64.5 | 12.4 | 23.0 |
| B8 | 98 | F | None | Survivor | 2 | 5.60 | 64.5 | 12.6 | 22.8 |
| B44 | 50 | F | None | Survivor | 2 | 2.40 | 63.2 | 12.5 | 24.2 |
| B77 | 63 | M | Anemia, UC, | Survivor | 2 | 3.90 | 61.6 | 11.4 | 26.8 |
| B73 | 91 | M | DM, HTN, HPLD,CVA | Survivor | 2 | 6.30 | 58.4 | 9.4 | 32.0 |
| B11 | * | F | HTN, HLD | Survivor | 2 | 5.20 | 48.9 | 5.5 | 45.5 |
| B40 | 64 | M | None | Survivor | 2 | 6.70 | 57.6 | 9.6 | 32.6 |
| B9 | 56 | F | HA, HLDP | Survivor | 2 | 9.10 | 64.0 | 14.3 | 21.6 |
| B23 | 43 | F | Depression | Survivor | 2 | 5.10 | 52.6 | 7.5 | 39.8 |
| B76 | 47 | M | None | Survivor | 2 | 7.40 | 59.3 | 11.7 | 28.8 |
| B56 | 50 | F | Bi-polar disorder, HTN | Survivor | 2 | 9.30 | 52.7 | 8.3 | 38.8 |
| A16 | 33 | M | None | Survivor | 2 | 5.10 | 55.8 | 10.7 | 33.3 |
| B80 | 57 | F | HTN, DM, HPLD, RSA | Survivor | 2 | 6.20 | 52.7 | 11.9 | 35.3 |
| B63 | 46 | M | HTN, CAD, HPLD | Survivor | 2 | 6.00 | 49.0 | 9.7 | 41.1 |
| B22 | 51 | M | DM, HTN, HPLD | Survivor | 2 | 7.20 | 44.3 | 10.1 | 45.5 |

**Table ST 4 (continued).**

| **Id**^1^ | **A**^2^ | **S**^3^ | **Co-morbidity(ies)*** | **Outcome** | **D**^4^ | **TLC**^6^ | **N %**^7^ | **M %**^8^ | **L %**^9^ |
| --- | --- | --- | --- | --- | --- | --- | --- | --- | --- |
| A22 | 76 | F | Cirrhosis | Survivor | 2 | 4.00 | 55.7 | 20.9 | 23.2 |
| A20 | 65 | F | CAD, DM,CHF, HTN | Survivor | 2 | 4.30 | 43.0 | 15.3 | 41.5 |
| A19 | 79 | F | AFIB, HTN | Survivor | 2 | 4.00 | 42.5 | 20.5 | 36.8 |
| A23 | 48 | M | DM | Survivor | 3 | 8.90 | 94.7 | 1.9 | 3.3 |
| B70 | 62 | M | ESRD, HD, HTN, DM | Survivor | 3 | 2.20 | 93.1 | 2.7 | 4.1 |
| B5 | 60 | M | OSA CPAP | Survivor | 3 | 7.40 | 88.6 | 2.3 | 8.9 |
| B82 | 61 | F | ASTHMA, DM | Survivor | 3 | 7.40 | 88.6 | 2.3 | 8.9 |
| B85 | 85 | M | ILD, DM, HTN, HLD, | Survivor | 3 | 8.50 | 88.6 | 2.3 | 8.9 |
| B35 | 69 | M | PIT adenoma, CAD | Survivor | 3 | 12.80 | 90.8 | 3.7 | 5.4 |
| B10 | 74 | F | DM, ILD, CHF | Survivor | 3 | 14.60 | 88.6 | 3.0 | 8.2 |
| B15 | 75 | M | HTN, lipids | Survivor | 3 | 8.50 | 85.1 | 2.7 | 12.0 |
| B69 | 51 | M | None | Survivor | 3 | 5.00 | 73.5 | 1.2 | 25.2 |
| B34 | 42 | F | Asthma, COPD, HTN | Survivor | 3 | 3.60 | 86.9 | 4.7 | 8.3 |
| B16 | 53 | M | Obesity | Survivor | 3 | 8.30 | 86.2 | 4.6 | 9.0 |
| A9 | 56 | M | None | Survivor | 3 | 12.10 | 88.4 | 6.0 | 5.4 |
| B64 | 69 | M | UC, HTN, HPLD, CAD | Survivor | 3 | 11.90 | 86.2 | 5.4 | 8.2 |
| B20 | 50 | F | Latent TB | Survivor | 3 | 8.00 | 85.7 | 5.3 | 8.9 |
| A7 | 63 | M | HTN, skin melanoma | Survivor | 3 | 11.40 | 86.5 | 5.8 | 7.5 |
| B29 | 79 | M | HYN, DM, HLPD, | Survivor | 3 | 6.30 | 87.1 | 6.5 | 6.2 |
| A10 | 71 | F | None | Survivor | 3 | 8.90 | 84.9 | 5.9 | 9.0 |
| A17 | 88 | F | COPD, dementia | Survivor | 3 | 8.10 | 87.7 | 8.5 | 3.7 |
| B19 | 61 | M | SZ, DVT, CAD, COPD | Survivor | 3 | 4.70 | 83.6 | 7.5 | 8.8 |
| B67 | 62 | M | Irritable bowel syndrome | Survivor | 3 | 5.30 | 81.5 | 6.7 | 11.7 |
| A13 | 75 | M | Gerd, HA, hypothyroidism | Survivor | 3 | 6.30 | 82.7 | 8.5 | 8.7 |
| B26 | 60 | F | Hypothyroidism | Survivor | 3 | 7.20 | 78.3 | 7.6 | 13.9 |
| B38 | 65 | F | None | Survivor | 3 | 9.50 | 76.0 | 6.6 | 17.2 |
| B83 | 56 | F | Glaucoma | Survivor | 3 | 5.90 | 78.4 | 8.2 | 13.2 |
| B13 | 70 | F | DM, IBS, RA | Survivor | 3 | 6.50 | 79.7 | 9.4 | 10.8 |
| B39 | 70 | M | AFIB, asthma, DM | Survivor | 3 | 11.30 | 80.4 | 10.0 | 9.4 |
| B50 | 92 | M | DVT, COPD, CKD | Survivor | 3 | 5.20 | 79.3 | 9.3 | 11.2 |
| B61 | 57 | M | HTN, DM2,CAD, OSA | Survivor | 3 | 8.40 | 77.7 | 8.6 | 13.5 |
| B21 | 51 | M | None | Survivor | 3 | 7.00 | 78.3 | 9.1 | 12.5 |
| B51 | 62 | M | Afib, DM2, HTN, CAD | Survivor | 3 | 8.00 | 79.8 | 10.4 | 9.7 |
| B9 | 56 | F | HA, HLDP | Survivor | 3 | 3.80 | 74.0 | 7.0 | 18.8 |
| B52 | 48 | M | None | Survivor | 3 | 6.90 | 74.1 | 7.1 | 18.6 |
| B78 | 68 | M | None | Survivor | 3 | 5.40 | 78.0 | 9.5 | 12.3 |
| B60 | 59 | M | None | Survivor | 3 | 8.90 | 77.3 | 9.6 | 13.0 |
| B33 | 51 | M | Asthma, HTN, OSA | Survivor | 3 | 4.70 | 75.5 | 9.1 | 15.3 |
| B44 | 50 | F | None | Survivor | 3 | 8.80 | 74.2 | 9.2 | 16.4 |
| B31 | 39 | F | DM | Survivor | 3 | 6.50 | 65.6 | 5.6 | 28.6 |

**Table ST 4 (continued).**

| **Id**^1^ | **A**^2^ | **S**^3^ | **Co-morbidity(ies)*** | **Outcome** | **D**^4^ | **TLC**^6^ | **N %**^7^ | **M %**^8^ | **L %**^9^ |
| --- | --- | --- | --- | --- | --- | --- | --- | --- | --- |
| A18 | 47 | M | Asthma, OSA | Survivor | 3 | 7.10 | 71.7 | 8.4 | 19.7 |
| A11 | 77 | M | OSA, CAD, HTN | Survivor | 3 | 7.30 | 76.1 | 12.1 | 11.7 |
| A24 | 53 | F | Asthma, HTN, HLDP | Survivor | 3 | 4.10 | 67.3 | 7.0 | 25.6 |
| B12 | * | M | Gout, HTN, | Survivor | 3 | 4.60 | 65.3 | 6.4 | 28.1 |
| B55 | 50 | M | HTN, CAD, OSA | Survivor | 3 | 10.30 | 73.9 | 11.3 | 14.6 |
| B18 | 50 | F | None | Survivor | 3 | 7.90 | 67.8 | 7.8 | 24.2 |
| B49 | 70 | M | OSA, BPN, Inc lft | Survivor | 3 | 7.10 | 71.2 | 9.7 | 18.9 |
| B47 | 62 | M | None | Survivor | 3 | 5.30 | 75.0 | 13.1 | 11.8 |
| B8 | 98 | F | None | Survivor | 3 | 9.00 | 70.0 | 10.1 | 19.7 |
| B62 | 61 | F | None | Survivor | 3 | 6.80 | 67.4 | 9.3 | 23.2 |
| B25 | 33 | M | None | Survivor | 3 | 2.80 | 60.5 | 6.4 | 33.0 |
| B73 | 91 | M | DM, HTN, HPLD | Survivor | 3 | 5.20 | 64.7 | 8.4 | 26.8 |
| B57 | 64 | F | OSA, anxiety | Survivor | 3 | 4.00 | 62.7 | 7.6 | 29.5 |
| B17 | 38 | M | Obesity, ex smoker | Survivor | 3 | 4.10 | 65.7 | 9.6 | 24.6 |
| B30 | 39 | F | Bi-polar disorder | Survivor | 3 | 6.30 | 64.5 | 9.3 | 26.1 |
| B28 | 50 | M | Kidney stone | Survivor | 3 | 9.70 | 66.5 | 10.8 | 22.6 |
| B58 | 58 | M | Panic attacks | Survivor | 3 | 5.30 | 68.1 | 12.9 | 18.8 |
| B37 | 54 | F | DM, asthma | Survivor | 3 | 3.80 | 67.1 | 12.2 | 20.5 |
| A8 | 57 | M | None | Survivor | 3 | 15.30 | 57.4 | 6.9 | 35.5 |
| B27 | 42 | F | HTN | Survivor | 3 | 7.90 | 63.7 | 11.0 | 25.1 |
| B54 | 38 | M | AVR, CVA, SZ, HTN | Survivor | 3 | 4.90 | 60.5 | 9.1 | 30.3 |
| B53 | 45 | M | None | Survivor | 3 | 8.60 | 58.5 | 10.1 | 31.2 |
| B41 | 59 | M | ESRD/HD, CAD | Survivor | 3 | 5.70 | 59.5 | 10.8 | 29.5 |
| B43 | 42 | M | COPD | Survivor | 3 | 5.70 | 60.4 | 12.6 | 26.8 |
| B42 | 56 | M | None | Survivor | 3 | 5.80 | 64.9 | 16.5 | 18.4 |
| B48 | 56 | M | None | Survivor | 3 | 5.80 | 64.9 | 16.5 | 18.4 |
| B40 | 64 | M | None | Survivor | 3 | 4.00 | 56.7 | 11.0 | 32.1 |
| B22 | 51 | M | DM, HTN, Apnea | Survivor | 3 | 4.90 | 47.9 | 6.7 | 45.3 |
| B56 | 50 | F | Bi-polar disorder | Survivor | 3 | 6.90 | 51.7 | 8.6 | 39.6 |
| A16 | 33 | M | None | Survivor | 3 | 6.30 | 58.2 | 13.5 | 28.1 |
| B46 | 56 | M | HPLD, Lyme hx | Survivor | 3 | 4.10 | 62.0 | 17.1 | 20.7 |
| B23 | 43 | F | Cardic abl svt | Survivor | 3 | 2.50 | 51.0 | 9.0 | 39.9 |
| B63 | 46 | M | HTN, CAD, HPLD | Survivor | 3 | 9.20 | 50.4 | 9.7 | 39.8 |
| A22 | 76 | F | Cirrhosis | Survivor | 3 | 5.10 | 60.4 | 19.2 | 20.3 |
| A21 | 64 | M | HRN, depression | Survivor | 3 | 4.40 | 48.7 | 13.2 | 37.9 |
| A20 | 65 | F | CAD, DM, AFIB,CHF | Survivor | 3 | 6.00 | 47.4 | 13.2 | 39.3 |
| B76 | 47 | M | None | Survivor | 3 | 4.00 | 49.1 | 17.9 | 32.8 |
| A19 | 79 | F | AFIB, HTN | Survivor | 3 | 3.50 | 46.3 | 17.4 | 36.2 |
| A14 | 92 | F | AFIB, HTN, PPM | Survivor | 3 | 2.60 | 52.3 | 25.8 | 21.8 |
| A7 | 63 | M | HTN, skin melanoma | Survivor | 4 | 11.40 | 88.4 | 4.4 | 7.1 |
| A17 | 88 | F | COPD, dementia | Survivor | 4 | 11.40 | 85.6 | 9.7 | 4.6 |
| A10 | 71 | F | None | Survivor | 4 | 8.90 | 78.5 | 6.4 | 14.9 |

**Supplementary Figure 1**

**Figure SF 1. Early clinical applications.** At day 1, one data point-wide lines of observations (1dpwlo) were observed, in both populations, when the three dimensionless variables described in Fig. 13 were plotted together (**A, B**). One or several data structures helped prognosticate outcomes. In the New Delhi dataset, 72.7 % (16/22) of all survivors were identified outside the cluster where non-survivors predominated (**A**). The same indicators discriminated, in the Jacksonville dataset, 36.4% (32/88) of all survivors (**B**). The Jacksonville dataset also revealed two data subsets only composed of survivors, who differed immunologically: the MC/N ratio differentiated subset A from B (**C**, **D**). Sixteen additional survivors were detected, in the Jacksonville dataset, when a third data structure was measured. Therefore, 54.5% (48 / 88) of all survivors were distinguished when three data structures were considered (**E**). When a fourth data structure was added, 5 more survivors were detected in the Jacksonville group, resulting in a cumulative detection of **60.2%** (53/88) of all survivors being identified at day 1 (**F**). While, in the New Delhi group, 93.8 [15/16] of all observations located within a distinct data subset were non-survivors, in the Jacksonville group only 23 % (5/22) of all non-survivors were found in the same data range (**G, H**).


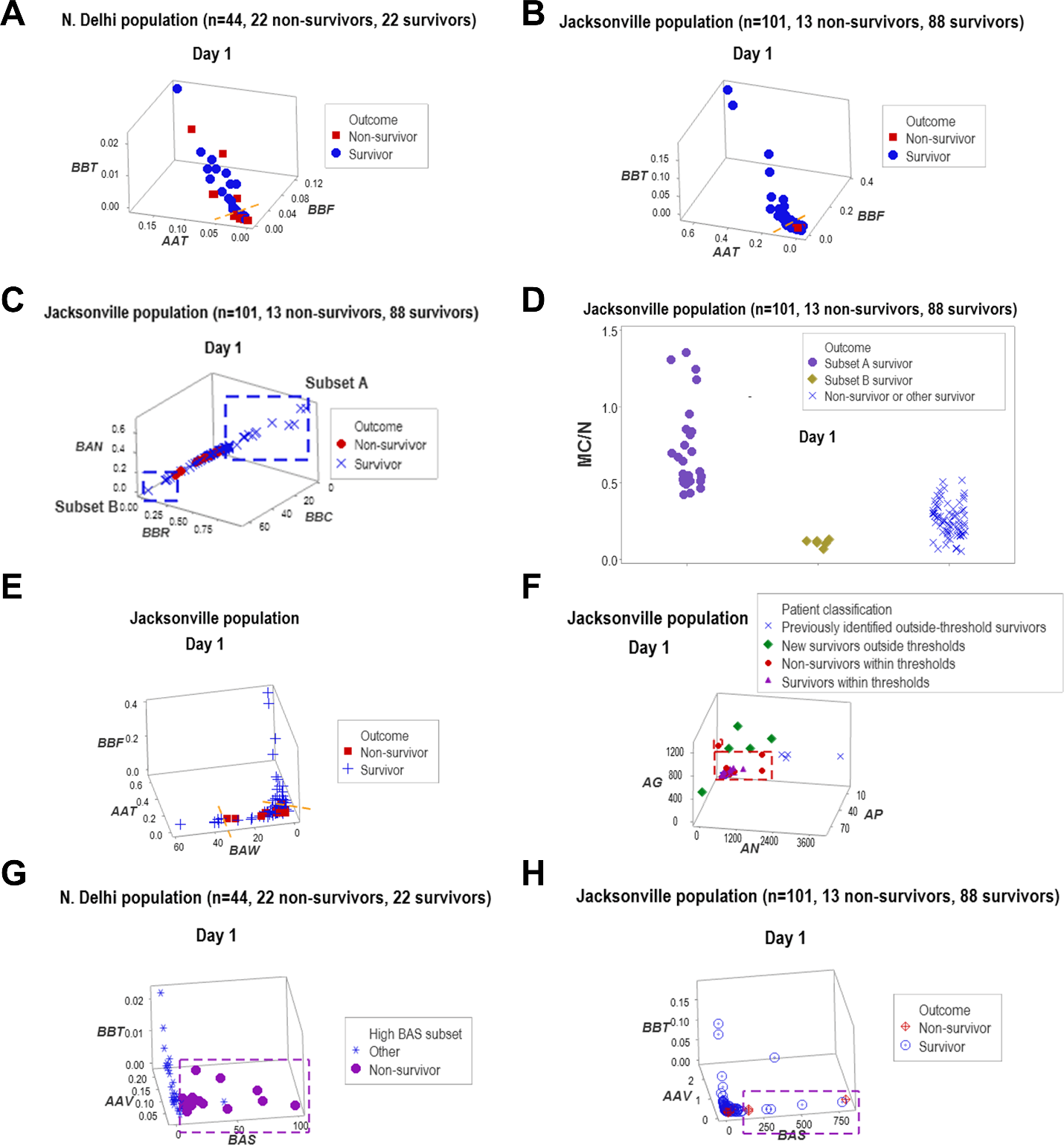

Supplement: Supplementary file 1 [file DataSheet_1.docx]
